# Determining the Global Economic Burden of External Health Effects of Food Consumption in 204 Countries and Territories

**DOI:** 10.3390/nu18030426

**Published:** 2026-01-28

**Authors:** Felix Seidel, Benjamin Oebel, Lennart Stein, Susanne Kleemann, Tobias Gaugler

**Affiliations:** 1 Faculty of Mathematics, Natural Sciences, and Materials Engineering, University of Augsburg, 86159 Augsburg, Germany; felix.seidel@uni-a.de; 2 Faculty of Business Administration, Nuremberg Institute of Technology, 90489 Nuremberg, Germany; lennart.stein@th-nuernberg.de (L.S.); tobias.gaugler@th-nuernberg.de (T.G.); 3 Faculty of Mathematics and Natural Sciences, University of Greifswald, 17489 Greifswald, Germany; susanne.stoll-kleemann@uni-greifswald.de; 4 Department of People and Society, Swedish University of Agricultural Sciences (SLU), 23456 Alnarp, Sweden

**Keywords:** sustainable nutrition, health costs, food consumption, nutritional inequality, dietary recommendations, public health, true prices, externalities

## Abstract

Background/Objectives: Every country and territory worldwide is affected by varying degrees of under- and overconsumption of food. A substantial share of the economic burden of unsustainable malnutrition arises from diet-related health impacts, although existing research has largely focused on environmental consequences. Methods: This study addresses this gap by combining cost-of-illness (COI) and True Cost Accounting (TCA) approaches, as well as Global Burden of Disease (GBD) data, to estimate external diet-induced health costs. A comprehensive database covering 204 countries and territories is established, quantifying health costs by disease category and dietary risk factor. Results: The results indicate that USD 1719.94 billion in annual global health costs are attributable to poor diets. This corresponds to an average burden of USD 211.08 per capita per year. Cardiovascular diseases (CVDs) constitute the largest share of costs, followed by diabetes mellitus (DM). In absolute and per capita terms, the United States contributes disproportionately. Regionally, North America bears 44.36% of the global monetary burden, while Oceania accounts for only 1.22%. The highest per-capita costs occur in North America, Europe, and Oceania. The most influential dietary risk factors are the overconsumption of processed and red meat, and the underconsumption of whole grains. A strong positive correlation is observed between diet-related health costs and national prosperity levels. Conclusions: This framework represents a novel approach to standardized and holistic valuation, providing a robust basis for deriving policy-relevant insights to inform sustainable nutrition strategies and advance the United Nations (UN) Sustainable Development Goals (SDGs), especially the second SDG, zero hunger.

## 1. Introduction

### 1.1. Sustainable Nutrition and Its Challenges

Obesity, high blood pressure, stroke, type 2 diabetes mellitus (T2DM), and cancer are major chronic diseases that can be influenced by diet [1]. At the same time, the emission of greenhouse gases (GHGs) during the production of food represents a significant burden for humanity and the environment [2]. It is evident that challenges extend beyond food production to include its distribution. This is underscored by the fact that approximately half of all deaths among children under five are attributable to food insecurity [3]. These factors are in direct opposition to the fundamental principle of sustainable nutrition, which combines environmental, health, economic, and social aspects [4]. Its overarching goal is to contribute to food security and health [5] (pp. 845–861). The underlying concept is characterized by a multidimensional approach, incorporating various approaches from different sources [5] (pp. 845–861).

The World Health Organization (WHO) underscores the significance of the interplay between the individual dimensions by emphasizing the need to promote healthy diets while respecting ecological boundaries, ensuring social and cultural acceptance, and guaranteeing economic accessibility [6]. Sustainable nutrition and its aspects are closely aligned with the SDGs. These goals, defined in the 2030 Agenda for Sustainable Nutrition and accepted by all UN member states in 2015 [7], are addressed to the global sustainability of society and represent a global commitment to the future of the world population [8]. Promoting and fulfilling the dimensions of sustainable nutrition thus contributes significantly to achieving the goals of the global community, especially the second, third, and twelfth objectives.

Both dietary sustainability and the SDGs are always jeopardized if external effects, such as damages not reflected in market prices [9], persist.

These so-called externalities are not confined to the production of food but occur throughout the entire value chain. The resulting welfare effects or changes in opportunities are not directly compensated or paid for, i.e., are not reflected in their market prices [10], and therefore lead to a distortion of the market. The aforementioned emissions and medical costs due to diseases constitute negative externalities, while concurrently, there are also positive ones, such as those that increase biodiversity or create jobs [11] (pp. 263–283). Education, which confers benefits to the individual recipient and society as a whole, is another example. The former can educate others, thereby benefiting them as well [12]. The result of negative food-related externalities is a reduction in the profitability and affordability of healthy and sustainable food [13]. External effects thus act as impediments to the establishment of sustainable food systems [14] (pp. 581–601). This is due to the fact that they promote the consumption of unhealthy diets, human rights violations, and the depletion of natural capital [14] (pp. 581–601). The aim of our work is to reduce these negative nutrition-related externalities by systematically quantifying the previously unrecorded diet-related health effects at the country level. This will make it possible, in a second step, to develop mechanisms for including these effects, such as those developed in our 2023 study for Germany [15], thereby moving food prices closer to their true societal costs.

### 1.2. Measurement of Health and Its Nutrition-Related Costs

In order to achieve the objective of our study as outlined above, methods and concepts for quantifying and monetizing health, as well as approaches for internalizing the resulting effects in prices, are required. These are briefly presented below. A structured summary is provided in Table 1. 

Gemmill-Herren et al. (2021) describe the concept of TCA, which is achieved through the employment of a multifaceted approach, encompassing environmental, human, social, and production-related factors [16]. The objective of TCA is to internalize external costs in the prices of products [9]. In the majority of cases, the focus of such studies is on quantifying and subsequently reducing the environmental costs induced by nutrition [19]. Jo (2014) explains the concept of COI studies, which are widely regarded as a pivotal technique within the healthcare sector [17] (pp. 327–337). As the term implies, COI studies are employed to estimate the monetary burden of diseases that threaten human health. As discussed below, depending on the study, this type of externality represents a large or even the largest part of the external effects of nutrition. Our study focuses on this burden, which has not yet been recorded in detail and in a globally uniform manner. This counteracts the aforementioned fact that the majority of the existing literature refers to the environmental component [19]. A distinction can be made between the direct, indirect, and intangible dimensions of costs [20]. The former includes direct healthcare costs, which can be divided into institutional inpatient care, institutional outpatient care, home healthcare, physician services, ancillary services, overhead costs, variable costs of utilities, medications, costs for devices and applications, drugs, diagnostic tests, treatment service costs, prevention service costs, rehabilitation costs, and costs for training and education. Conversely, there are also direct non-healthcare expenses to consider, including social services, program evaluation, repair of property destruction, transportation, time expenditure, childcare, and housekeeping, as well as legal costs. The second dimension comprises indirect costs due to productivity losses, foregone leisure time, and informal care [17] (pp. 327–337). Intangible costs, i.e., costs due to pain and suffering [21], are often not taken into account in corresponding studies, as it is difficult to quantify them in monetary terms [22] (pp. 695–704). In parallel, the GBD study records the burden of diseases. It signifies the most substantial and exhaustive endeavor to comprehensively quantify health loss across diverse geographical locations and temporal periods, with the objective of enhancing health systems and eradicating inequalities [23]. In terms of nutrition, the promotion of health, social, and economic dimensions of sustainable nutrition is of primary importance. The corresponding GBD Compare tool facilitates the undertaking of a diversity of possible settings, thus enabling the collection of numerous statistics on the burden of disease of various diseases. For instance, it is possible to differentiate between dietary risk factors and individual countries and territories [18]. The tool thus offers a holistic and comprehensive overview of the burden of various diseases and is also used in the course of our study. Concerning the diseases caused by diet [24], it was found, according to the GBD study, that CVDs, DM, and neoplasms are mostly induced [24]. The remaining diseases account for a negligible proportion of the burden of disease caused by malnutrition [25] (pp. 1958–1972). If these additional factors were taken into account, the effort involved would increase significantly in comparison to the benefits, which is why they are not considered in our study. For each additional disease included, further uncertainties would occur, particularly in the case of missing data. The latter problem of information gaps is especially likely to arise with this marginal proportion, as less funding and therefore fewer studies are to be expected due to the lower burden involved [26].

After reviewing the TCA and COI approaches as well as the GBD study, we present an estimate of the costs associated with a balanced diet and what monetary expenditure would be required to eliminate global hunger. Then, it is explained which studies exist on the costs of an unsustainable and unhealthy diet and how these connect to the approaches mentioned above. Table 2 summarizes all the studies listed below.

The first study introduces the concept of food safety and determines the costs of a healthy diet by continent and income based on current food prices and the FAO Food Prices Index. From a global perspective, 3.96 purchasing power parity (PPP) dollars are needed for a balanced diet [27]. The costs vary depending on the continent and sub-region, with the highest in Eastern Asia (PPP dollars 5.34) and the lowest in Northern America (PPP dollars 2.96). Innovative financing options for individual countries are also presented, which should contribute to the fulfilment of the SDGs [27]. Another study estimates the cost of a balanced diet for the year 2017 at between USD 0.71 and 1.77, with the price increasing if animal-based foods such as red meat, poultry, and fish are included [24]. In addition, the study estimates the cost of eliminating global hunger at USD 265 billion per year based on a partial-equilibrium model, of which the majority of USD 198 billion is allocated to investments for the poor and the remaining USD 67 billion to social protection [24]. As in our study, further research combines COI and GBD data to estimate global health and, in addition, environmental costs for 2030 [28]. The health costs amount to approximately USD 1.30 trillion, while environmental implications incur USD 1.70 trillion [28]. Unlike our study from 2023 [15] and also this current work, connectors for COI and GBD data are used based on meta-analyses of prospective cohort studies to estimate the monetary burden [28]. Thus, there is no direct offsetting of GBD with COI data. In another study, the hidden costs of food in 2020 are estimated based on the TCA approach at USD 11.60 trillion, of which USD 8.10 trillion represents health costs [29]. As already mentioned, COI and GBD data are utilized in our 2023 study to determine health costs in Germany in 2022. For the first time, the underlying methodology directly links disease-related data with its costs at the country level. This is achieved by offsetting the costs of diseases against the relative disability-adjusted life years (DALYs) caused by certain risk factors [15]. The latter quantifies the impact of a disease on life expectancy by assuming that the healthy years of life a human is born with are reduced by either the years lived with the disease (YLD) or by the years lost due to premature death (YLL) [30]. This fine-grained differentiation according to dietary risk factors and diseases has made it possible for the first time to calculate country- and food-specific health costs and derive recommendations from them. Thus, the total diet-induced health costs in Germany in 2022 are estimated at EUR 50.38 billion, which corresponds to EUR 601.48 per capita [15].

Existing research on the economic implications of nutrition-related externalities has largely focused on environmental impacts. Despite the assumption, based on current data, that the health implications of inadequate diets constitute a significant financial burden for society [28,29], and that these are not incorporated into the market prices of food, the subject has received scant attention in academic research. The health costs of nutrition are typically recorded from a global perspective, or alternatively, they are categorized according to certain indicators. In either case, a group of different countries and territories is always considered. To date, there has been only one study by Seidel et al. (2023) that has determined the costs of certain diet-related risk factors from a fine-grained perspective for a specific country [15]. This highlights the lack of studies examining diet-related health costs from a global perspective and, at the same time, breaking them down by country and territory and risk factors. Our study aims to achieve this goal by employing the TCA and COI approaches previously referenced, in conjunction with data obtained from the GBD study. In the first step, the standardized health costs of CVDs, DM, and neoplasms for the year 2024 are systematically determined before being converted to nutrition in the second step using the aforementioned DALYs. The objective of our study is to ascertain the costs of external health impacts of food consumption for 204 countries and territories and to discuss the resulting implications for sustainable nutrition and the goals of the global community. This work thus provides an answer to the questions of how the diet-induced health costs can be calculated in a global scope for 204 countries and territories, and what recommendations can be derived from this for a healthier diet worldwide. In the following section, the methods and approaches developed in our study for the uniform determination of country-specific COI data and the health costs resulting from the use of information from the GBD study are presented. Afterwards, the sources and their uncertainty are briefly discussed before presenting the outcomes. After the results have been evaluated and discussed, a final conclusion is drawn.

## 2. Materials and Methods

### 2.1. Method Outline

In this section, the procedure for standardizing heterogeneous data is first described. The subsequent chapter will address the methodology for estimating the COI of countries and territories that encounter partial or missing data. The determination of partial data is achieved through the utilization of a factor that describes the respective disease, while missing values are determined using a score-based comparison. Detailed information on the former is listed in Supplementary Materials File S1/Section S1, while the procedure for the latter in the event of missing data is listed in Supplementary Materials File S1/Section S2. In the score-based comparison, similar countries and territories are ascertained by taking into account the respective share of healthcare costs in economic output for direct costs, the relative productivity for indirect costs, as well as the relative burden of the disease to be estimated. A factor is then used to determine the direct costs, after which the indirect part is calculated by relation. These data are then utilized to obtain country- and risk-factor-specific health costs for 204 countries and territories that currently represent externalities. Supplementary Materials File S1/Section S3 addresses the calculation, estimation, and source data. Finally, any uncertainties and limitations arising in this process are identified in Supplementary Materials File S1/Section S4.

### 2.2. Method in Short

Prior to the delineation of the methodology for quantifying the direct and indirect costs of specific diseases and the subsequent calculation of per-country health costs, it is imperative to establish the following subsections.

**Figure 1:**
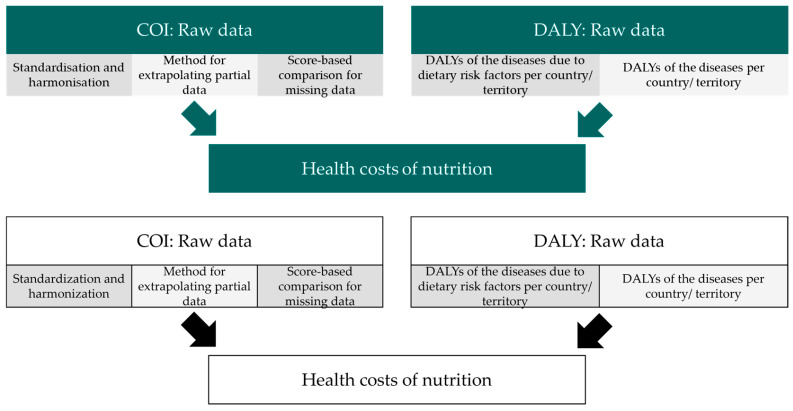
Visualization of the methodological workflow for estimating health costs of nutrition, integrating cost-of-illness data and disability-adjusted life years.

Firstly, it is explained how data from different studies can be made comparable and how heterogeneous data from individual studies are modeled. Section 2.2.2 comprises the estimation of the total or partial COI of countries and territories, for which only partially complete data are available from studies. A procedure is subsequently formulated for the estimation of direct and indirect costs in instances where the data are unavailable. Finally, a methodology for the extraction of the share of COI attributable to specific dietary risk factors via DALYs is hereby presented. The entire procedure is illustrated in Figure 1. This approach creates a database that, on the one hand, contains the monetary burden of the diseases under consideration, and, on the other hand, links this to nutrition through the use of DALYs. Through a finely granular conversion, diet-induced health costs can be viewed differentiated by country and territory, risk factors, and diseases. This represents the first study to carry out this analysis from a global perspective, thus providing an important basis for future economic research on health impacts.

#### 2.2.1. Standardization of Heterogeneous Data and Harmonization of the Underlying Currencies

In the event that direct (*c_dir_*_,*x*,*d*,*y*,*cur*_) and/or indirect costs (*c_ind_*_,*x*,*d*,*y*,*cur*_) are available for the respective country or territory (*x*) and disease (*d*) under consideration in a given year (*y*), these are converted uniformly from the original currency (*cur*) into USD in the first step. The USD exchange rate (*FX_y_*_,*USD*,*cur*_) that applied in the respective year (*y*) is used, and the USD is thus established in relation to the respective other currency. In instances where a single value is not available for this, the average of the monthly exchange rates (*FX_m_*_,*USD*,*cur*_) is utilized.

(1) $$c_{dir,x,d,y,USD}=c_{dir,x,d,y,cur}\times {FX}_{y,USD,cur}$$

(2) $$c_{ind,x,d,y,USD}=c_{ind,x,d,y,cur}\times {FX}_{y,USD,cur}$$

(3) $${FX}_{y,USD,cur}=\sum {FX}_{m,USD,cur}÷12$$

Following the transformation of direct and indirect costs into the uniform currency USD, adjustments are made to these costs, both inflationary and demographic, to the year 2024. Thereafter, the standardized total costs (*C_x_*_,*d*,2024,*USD*_) are calculated. The consumer price indices for the USA from 2024 (*CPI_USA_*_,2024_) and the original year (*CPI_USA_*_,*y*_) are utilized for the former. For the latter, population data for the corresponding country or territory from both years (*p_x_*_,2024_; *p_x_*_,*y*_) are used. Given that the currency was already converted to USD, this necessitates the utilization of US data, while the demographic adjustment requires specific data for each country and territory.

(4) $$C_{x,d,2024,USD}={(c}_{dir,x,d,y,USD}+c_{ind,x,d,y,USD})\times \frac{{CPI}_{USA,2024}}{{CPI}_{USA,y}}\times \frac{p_{x,2024}}{p_{x,y}}$$

In the event of the rounding of the costs of the individual countries and territories in a study being scaled in such a manner that a value of zero is the result, further calculation is required. The proportion of another variable, in this case gross domestic product (GDP), is used to determine the actual COI. We multiply it by the GDP of the country or territory from the corresponding year to obtain the health costs.

(5) $$C_{x,d,y,cur}=\frac{C_{x,d,y,cur}}{{GDP}_{x,y}}\times {GDP}_{x,y}$$

The resulting value is then standardized according to the system already described and adjusted for inflation and demographics for the year 2024.

Finally, the possibility is considered in which data on the COI of a disease are available in a study, but only as a share of another variable. In this case, this variable is GDP, which itself originates from an aggregation over a certain period. The following computation incorporates the assumption of exponential growth modeling, which is derived from the growth factor (*r*) of another variable (*V*), the level of which is certain at the start (*sy*) and end (*ey*) of the period. The calculation is based on the country’s GDP per capita (*cap*) in USD (*GDP_x_*_,*cap*,*USD*_) and its population in 2024 (*p_x_*_,2024_). The multiplication of both values is identical to the use of total GDP above.

(6) $$r\left(V\right)={\frac{V_{ey}}{V_{sy}}}^{\frac{1}{1+ey-sy}}-1$$

(7) $$C_{x,d,2024,USD}={GDP}_{x,cap,USD}\times p_{x,2024}\times \frac{\frac{C_{x,d,ey-sy,USD}}{{GDP}_{x,ey-sy,USD}}\times r\left(V\right)\times (1+ey-sy)}{\left({1+r\left(V\right)}^{1+ey-sy}\right)-1}\times {\left(1+r\left(V\right)\right)}^{2024-sy}$$

#### 2.2.2. Method for Calculating the COI for Partial Costs

**Figure 2:**
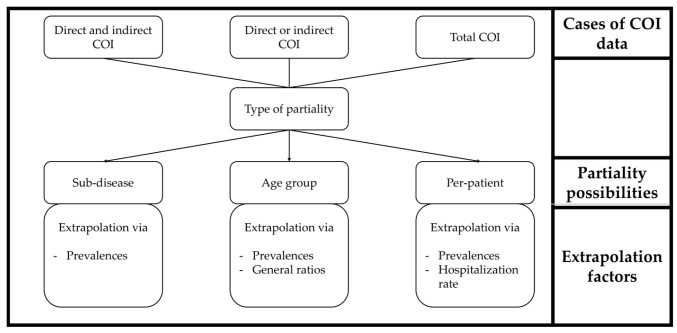
Partiality of existing cost-of-illness data by type and respective extrapolation factors.

This subsection addresses the scenario in which comprehensive health cost data are not available at the national or territorial level, but only partial information is accessible. Figure 2 visualizes the method developed for this purpose. The top hierarchy shows the three cases of existing COI data discussed below. Partiality then results from the possibilities also illustrated, for which corresponding extrapolation factors are required in order to obtain complete information. The following elaboration will differentiate between the aforementioned three possible cases. In the first case, data are accessible on both the direct and indirect COI of a given country. In the second case, information is exclusively available either on the direct or on the indirect costs. In the third and final case, there is only certainty about the total costs, i.e., the sum of direct and indirect costs, whereby no more precise differentiation is made. In all three cases, the partiality of the costs is attributable to their correspondence to a subcategory of the totality under consideration. The first possibility of partiality in every case is the existence of data on the costs of sub-diseases, i.e., a part of the actual disease to be determined. The second possibility describes the existence of data for a specific age group, without including the rest of the population. The third option deals with per-patient costs, whereby only the per-capita costs of certain patients are determined. In this context, there may be various further subdivisions, such as corresponding costs for top categories of diseases or distributions according to the type of healthcare facility.

The extrapolation is invariably conducted employing prevalences, which define the total number of cases occurring at a certain time [31]. The resulting values require subsequent standardization as well as inflationary and demographic adjustment according to the system that was previously presented.

The underlying rationale is that prevalence is directly related to the corresponding costs. In the first possibility described above, the total costs of the disease can be determined by comparing the number of people with the sub-disease to the number of people with the disease. The second option follows the same logic, but for a specific age group. In order to scale data from multiple sources in a meaningful way, the general direct and indirect cost components of the disease are also required. In the third possibility, where per-patient costs are available, additional information is required for both types of costs. For direct costs, the prevalence rates are offset against the hospitalization rate for the country or territory. This indicates the proportion of patients who use healthcare facilities and services, as direct costs are only incurred for these. Indirect costs are multiplied by the number of patients who are not retired and are working. The detailed procedure for all cases and possibilities is described in Supplementary Materials File S1/Section S1.

In order to determine the missing COI proportions in the second and third cases, the score-based comparison described in the following section is applied.

#### 2.2.3. Score-Based Comparison for Estimating Missing COI Values

**Figure 3:**
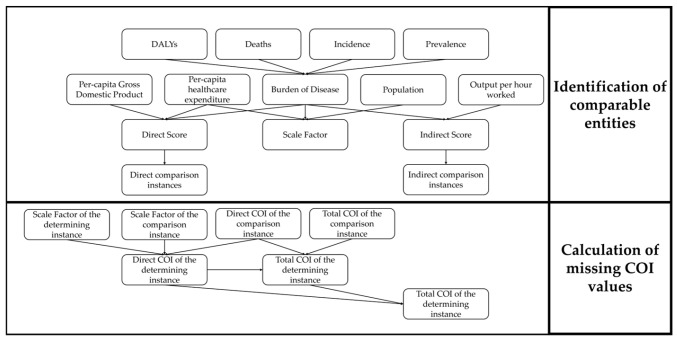
Visualized process of the score-based comparison.

The detailed process of the score-based comparison is shown in Figure 3. In the first step, comparable instances are determined using scores for both missing direct and indirect costs. In addition, a scale factor is calculated for each individual country and territory, which is then required for the direct costs. The missing values are then ascertained on the basis of the identified comparison objects, thus determining complete COI values.

In instances where direct or indirect costs are not available for a given country or territory, these values are estimated through comparison with other similar values. The factors considered depend on the COI aspect to be calculated. It is important to note that the corresponding known COI values were already adjusted for inflation and demographics for the year 2024. This means that the values resulting from the comparison can be directly transferred.

A total of five factors are taken into account when calculating direct costs. The first is the share of PPP-adjusted per-capita healthcare expenditure (*H_PPP_*_,*x*,*cap*,*y*_) in the PPP-adjusted per-capita GDP (*GDP_PPP_*_,*x*,*cap*,*y*_) of the respective country or territory (*K_rel_*_,*x*,*y*_). This value is a measure of the direct burden of disease in a country as a proportion of the economic output spent on healthcare.

(8) $$K_{rel,x,y}=\frac{H_{PPP,x,cap,y}}{{GDP}_{PPP,x,cap,y}}$$

The second factor for calculating the direct costs is the proportion of the aforementioned DALYs of the analyzed disease in the respective country or territory (*DALY_x_*_,*d*,*y*_) in relation to the total value of all diseases (*DALY_x_*_,*y*_). In order to scale this burden for all countries and territories in a meaningful way, the relative value is used.

(9) $${DALY-share}_{x,d,y}=\frac{{DALY}_{x,d,y}}{{DALY}_{x,y}}$$

The third factor delineates the ratio of deaths from the respective disease (*Deaths_x_*_,*d*,*y*_) to the total deaths from diseases in the country or territory (*Deaths_x_*_,*y*_). Consequently, it can be regarded as a metric for the comparative mortality rates of the disease.

(10) $${Deaths-share}_{x,d,y}=\frac{{Deaths}_{x,d,y}}{{Deaths}_{x,y}}$$

The fourth factor describes the relative incidence of the disease (*I_x_*_,*d*,*y*_) in relation to the total incidence (*I_x_*_,*y*_). The term is defined as a measure of the rate at which something happens [32]. Consequently, it can be interpreted as the frequency of new cases within a specified time period.

(11) $${I-share}_{x,d,y}=\frac{I_{x,d,y}}{I_{x,y}}$$

The final factor denotes the proportion of the prevalence of the analyzed disease (*P_x_*_,*d*,*y*_) in the total prevalence (*P_x_*_,*y*_) in the considered country or territory. It is described as the fact that something happens with high frequency or occurs very often [33]. In contrast to incidence, both old and new values are taken into account. While the cure or death of a patient does not influence the incidence, the prevalence decreases in these cases [34].

(12) $${P-share}_{x,d,y}=\frac{P_{x,d,y}}{P_{x,y}}$$

In summary, factor one represents the direct, relative economic weight of disease in a given country or territory. Factors two to five accumulate to represent the relative disease burden of the specific disease in question. The combination of these aspects enables the determination of the countries in which diseases are similarly prevalent (factors 2–5). Simultaneously, it ascertains whether the proportionate health expenditure, adjusted for purchasing power, is in a comparable ratio (factor 1). The relative healthcare costs are given a weighting of 0.3, the DALY, incidence, and prevalence ratios each have a share of 0.2, and the relative deaths have a weighting of 0.1. The emphasis is thus placed on a thorough economic and medical analysis, with the death factor being assigned a lower priority due to its minimal influence on healthcare expenditure.

(13) $$\begin{array}{lll} {Score}_{dir,x,d}=0.3 & \times & K_{rel,x,y}+0.2\times {DALY-share}_{x,d,y}+0.1\times {Deaths-share}_{x,d,y}+0.2\times {I-share}_{x,d,y}\\ & + & 0.2\times {P-share}_{x,d,y} \end{array}$$

A distance matrix is formed from the resulting scores, which shows the absolute difference between them. Consequently, each country and territory for which data are available can, therefore, be compared with every other one. The ten most suitable comparison objects are then determined for each country and territory by calculating the minimum of the values resulting from the subtraction of the scores. The prioritization rules for the subsequent selection of comparison countries are as follows. For the purpose of our study, the top ten countries and territories in terms of minimum distances are to be considered. Priority is given to primary data from external sources. Furthermore, care is also taken to ensure that, where possible, geographical similarities such as spatial proximity or geographical structures are taken into account. Finally, the plausibility of the calculated data is checked by comparing its COI level with other diseases’ levels. For example, it would most likely not make sense if the estimated health costs of one disease in one country or territory were only a small fraction of another disease there.

The determination of a scale factor (*SF_x_*_,*d*_) enables the comparison of two countries or territories with each other. A composite index is utilized to compare analogous countries and territories, incorporating demographic, economic, and disease-burden metrics. In the case of the former, the corresponding population size in the target year 2024 is used. For the second, the current, non-PPP-adjusted per-capita healthcare expenditure of the country or territory (*H_x_*_,*cap*,*y*_). For the latter, the current burden of disease (*Burden-share_x_*_,*d*,*y*_) is employed. The exposure to illness is composed of the four share-based factors of DALYs, deaths, incidence, and prevalence. In this instance, it is imperative to consider the unadjusted healthcare expenditure, as the COI to be determined should also be expressed in the real currency USD and not in the artificial currencies PPP dollars or International dollars (INT$).

(14) $${SF}_{x,d}=p_{x,2024}\times H_{x,cap,y}\times {Burden-share}_{x,d,y}$$

(15) $$\begin{array}{ll} {Burden-share}_{x,d,y} & \\ & =0.3\times {DALY-share}_{x,d,y}+0.1\times {Death-share}_{x,d,y}\\ & +0.3\times {I-share}_{x,d,y}+0.3\times {P-share}_{x,d,y} \end{array}$$

The weighting of the individual factors of the burden share is consistent with the weighting utilized in the calculation of scores. In this calculation, the DALY, incidence, and prevalence shares are assigned higher weights than previously, at 0.3, while the deaths share equals the same weight at 0.1. The purpose of this scale factor is to standardize the total healthcare expenditure of a country or territory with the corresponding disease burden of the selected disease. Consequently, it serves as an effective indicator for the direct COI and forms the foundation for subsequent comparison. The validity of the values calculated using the scale factors is checked by estimating those for countries or territories for which data are already available.

In order to undertake a comparison between two countries or territories, the most suitable comparison object is selected in the distance matrix according to the previously outlined procedure. The scale factor of the country or territory to be estimated (*SF_est_*_,*x*,*d*_) is then divided by the scale factor of the one to be compared (*SF_comp_*_,*x*,*d*_). The direct, known COI (*c_dir_*_,*comp*,*x*,*d*,2024,*USD*_) is then multiplied by this ratio to obtain the unknown, direct COI to be estimated (*c_dir_*_,*est*,*x*,*d*,2024,*USD*_).

(16) $$c_{dir,est,x,d,2024,USD}=\frac{{SF}_{est,x,d}}{{SF}_{comp,x,d}}\times c_{dir,comp,x,d,2024,USD}$$

The result of this methodology is the calculation and estimation of the direct COI of countries and territories where no data are available. In the first step, comparison objects are determined using scores and a resulting distance matrix, prior to the calculation of previously unknown health costs using a scale factor in the second step.

Having explained in the preceding subsection which criteria and factors are used to estimate unknown direct health costs, the corresponding indirect part will now be examined. In order to compare countries or territories in terms of direct health costs in a meaningful way, appropriate metrics are used to quantify the monetary weight of diseases. This allows for the comparison of entities that have a similar direct burden from diseases. In order to compare indirect health costs, the direct share must be adjusted. To achieve this, the monetary aspect is replaced by a productivity factor so that countries and territories with a similar indirect burden can be compared.

Firstly, the individual aspects of the score for determining the distance matrix will now be discussed in the familiar system. The first of the five factors is the aforementioned measure of the productivity of workers in the respective country or territory. It determines the efficiency with which labor input and other factors are implemented in the production process [35]. The corresponding measure is the output per hour worked (*OPHW_x_*) in the respective country or territory (*x*), whereby GDP adjusted for purchasing power is used to determine it. In order to make this value comparable across all countries and territories in a global context, the maximum value (*OPHW_max_*) is determined, and all data are set in relation to it, resulting in relative productivities (*OPHW-share_x_*).

(17) $${OPHW-share}_{x}=\frac{{OPHW}_{x}}{{OPHW}_{max}}$$

The remaining four factors correspond to burden of illness determinants that were previously utilized to calculate the direct COI values.

In summary, the combination of OPHW and the four burden of disease aspects represents a suitable synthesis of productivity and presence of the illness in order to estimate the indirect part of COI. This is made up of productivity losses, mortality, morbidity, impairments, and absences from work [17] (pp. 327–337). The weightings are set in accordance with the system for estimating direct costs. The productivity score is assigned a weight of 0.3, the DALY-, I-, and P-shares each a weight of 0.2, and the deaths share a weight of 0.1. This approach enables the scaling of the indirect burden in relation to the presence of the respective disease in the country or territory under consideration, thus facilitating a comprehensive economic and medical analysis.

A distance matrix is then formed from the individual scores. The ten countries and territories that are best suited for comparison with the selected one are determined. The focus is directed towards a reduction in disparity and the identification of geographical affinities.

Indirect costs do not require a scale factor in the same sense as direct costs. In this instance, the ratio of the known total costs (*C_comp_*_,*x*,*d*,2024,*USD*_) and the direct costs (*c_dir_*_,*comp*,*x*,*d*,2024,*USD*_) of the reference country or territory is formed and then offset against the direct costs of the country or territory to be estimated (*c_dir_*_,*est*,*x*,*d*,2024,*USD*_) in order to determine its total costs (*C_est_*_,*x*,*d*,2024,*USD*_).

(18) $$C_{est,x,d,2024,USD}=\frac{C_{comp,x,d,2024,USD}}{c_{dir,comp,x,d,2024,USD}}\times c_{dir,est,x,d,2024,USD}$$

The difference between total costs and direct costs is then calculated to obtain the indirect COI of the disease (*c_ind_*_,*est*,*x*,*d*,2024,*USD*_).

(19) $$c_{ind,est,x,d,2024,USD}=C_{est,x,d,2024,USD}-c_{dir,est,x,d,2024,USD}$$

The result of this methodology is the calculation and estimation of the indirect and total COI of countries and territories for which no data are available. In this instance, too, scores and a distance matrix are utilized to ascertain comparison objects, which are then employed to estimate the missing values. The underlying logic of this approach is that if productivity and disease burden are comparable, it can be deduced that the proportion of indirect costs in total costs will also be analogous for the countries or territories under consideration.

If certain information is not available for countries and territories, an adjusted calculation using modified scores and scaling factors is required, as described in detail in Supplementary Materials File S1/Section S2.

#### 2.2.4. Calculating the Health Costs of Nutrition

In the preceding subsections, methodologies and procedures were developed for the uniform standardization of existing, heterogeneous data with different currencies. In addition, the calculation or estimation of partially or completely missing data was addressed. For the latter, a score-based comparison was established to estimate the direct and indirect COIs for countries and territories separately on the basis of different factors. The methodology underlying our study to evaluate the health costs of malnutrition is presented below. This approach can be used to convert the pure health costs of diseases to the risk factors of nutrition. The exhibited formulas and correlations are derived from the study by Seidel et al. (2023), which quantifies the corresponding health effects of nutrition for Germany and develops a methodology that can be applied to all countries and territories [15].

In the first step, a factor is required to standardize the total health costs of the individual diseases to the proportion that is exclusively due to nutrition. The DALY rate (*q_x_*_,*r*,*d*_) is calculated from the ratio of the total DALYs in the respective country or territory (*DALY_x_*_,*d*_) and the DALYs that are incurred due to dietary risk factors (*r*) in the form of the corresponding disease (*DALY_x_*_,*r*,*d*_). This rate serves to illustrate the comparative burden of the respective disease in relation to the risk factors that contribute to its development.

(20) $$q_{x,r,d}={DALY}_{x,r,d}÷{DALY}_{x,d}$$

The health costs of the diseases are then calculated based on the individual risk factors of the countries and territories (*HC_x_*_,*r*,*d*,2024,*USD*_) by multiplying the COI of the respective diseases by the corresponding DALY rates.

(21) $${HC}_{x,r,d,2024,USD}=C_{x,d,2024,USD}\times q_{x,r,d}$$

By dividing these costs by the respective population of the country or territory in 2024, we obtain the per-capita costs of the individual dietary risk factors that are reflected in the selected diseases.

(22) $${HC}_{x,r,d,cap.2024,USD}=\frac{{HC}_{x,r,d,2024,USD}}{p_{x,2024}}$$

This represents the conclusion of the overall method. The proportion of dietary risk factors was inferred from the general costs of individual diseases using the DALY rates.

While the model is explained in theory, Supplementary Materials File S1/Section S3 discusses the input data for quantification and monetization. First, general input data are explained [36,37,38,39,40,41,42,43,44,45,46,47], followed by the sources used to determine the monetary burden of the diseases [48,49,50,51,52,53,54,55,56,57,58,59,60,61,62,63,64,65,66,67,68,69,70,71,72,73,74,75,76,77]. The studies used to estimate the hospitalization rate are also listed here [78,79,80,81,82,83]. In addition, the source used to obtain the per-capita GDP data for determining the COI for neoplasms is provided [84]. Finally, the input required to determine the burden of the diseases is listed. Supplementary Materials File S1/Section S4 additionally discusses the uncertainties and limitations arising from the method and the input data [85,86,87,88,89].

## 3. Results

This section summarizes the empirical results of this study and is structured into several analytical subsections. Section 3.1 quantifies the total and per-capita economic burden of cardiovascular diseases, diabetes mellitus, and neoplasms across 204 countries and territories. Section 3.2 focuses on diet-induced health costs, presenting their overall magnitude and disease-specific composition, while Section 3.2.1 compares these costs across countries and territories. Section 3.2.2 and Section 3.2.3 further disaggregate nutrition-related health costs by continent and by individual dietary risk factors, respectively. Finally, Section 3.2.4 compares diet-induced health costs with GDP.

### 3.1. The Economic Burden of CVDs, DM, and Neoplasms in 204 Countries and Territories

All of the total and per-capita costs of the individual countries and territories, broken down by disease and direct and indirect share, can be found in the table ”Direct, indirect, and total COI” in Supplementary Materials File S2. An aggregated list with only the total costs per disease and country or territory can be accessed in the ”Total COI” table in Supplementary Materials File S2.

In total, CVDs, DM, and neoplasms account for approximately USD 4794.33 billion in total and USD 588.39 per-capita health costs on a global scale. Of this, USD 2162.79 billion (45.11%) is attributable to CVDs, USD 1982.18 billion (41.34%) to DM, and USD 649.37 billion (13.54%) to neoplasms.

In 2024, the USA incurs by far the highest total economic burden of CVDs (USD 773.54 billion), while Switzerland records the highest per-capita costs (USD 4047.23) compared with a global average of USD 265.43.

For DM, the USA likewise bears the greatest total costs (USD 721.01 billion), whereas Monaco exhibits the highest per-capita burden (USD 2215.92) relative to the global average of USD 243.27.

Neoplasms follow a similar pattern, with the USA generating the largest overall costs (USD 227.57 billion) and Monaco showing the highest per-individual costs (USD 2837.91), far above the global average of USD 79.70.

Table 3 shows the total and per-capita health costs of the countries and territories with the largest populations.

### 3.2. The Economic Burden of Diet-Related Diseases in 204 Countries and Territories

A detailed list of the health costs of nutrition presented in the following can be found in the table ”Diet-induced health costs” in Supplementary Materials File S2; there, the costs per country and territory are displayed and categorized according to the respective disease and the dietary risk factor causing it.

**Figure 4:**
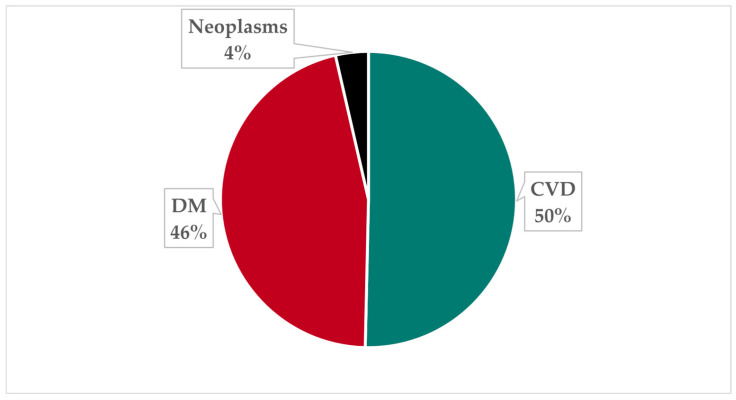
Shares of the cost-of-illness of cardiovascular diseases, diabetes mellitus, and neoplasms in global diet-induced health costs for 2024.

Globally, approximately USD 1719.94 billion in total and USD 211.08 in per-capita health costs are incurred as a result of malnutrition in 2024. Just over half of this total value, corresponding to USD 866.00 billion (50.35%), is in the form of CVDs, while USD 792.21 billion (46.06%) is due to DM. A mere USD 61.72 billion (3.59%) of the external health costs of nutrition are attributable to neoplasms. The proportions determined are shown in Figure 4.

Underconsumption is responsible for slightly more diet-induced health costs (USD 894.98 billion; 52.04% of the health costs) than overconsumption (USD 824.96 billion; 47.96% of the health costs).

The proportion of health costs of the three diseases induced by poor nutrition in the total COI is 35.87%. With regard to CVDs and DM, the former results in slightly more and the latter in slightly less than 40.00% of the respective costs due to poor nutrition. The proportion for the former is 40.04%, while for the latter it is 39.97%. Neoplasms are not generally associated with poor nutritional status; rather, they are triggered by other factors. It is estimated that 9.51% of the annual costs associated with this disease are attributable to excessive and deficient consumption of food.

#### 3.2.1. Comparison of Diet-Induced Health Costs in 204 Countries and Territories

The highest health costs due to poor diets are incurred in the USA with USD 689.90 billion (40.11% of total costs), followed by China with USD 130.68 billion (7.54% of total costs), India with USD 87.54 billion (5.08% of total costs), Germany with USD 84.81 billion (4.93% of total costs), and Japan with USD 62.44 billion (3.59% of total costs). In total, these five countries induce USD 1053.55 billion (61.26% of total costs), while the remaining 199 countries and territories incur USD 666.39 billion in diet-induced health costs (38.74% of total costs).

The results show that the bottom half of countries and territories, measured by health costs of nutrition, contribute USD 16.04 billion in total. This corresponds to a global share of less than one percent (0.93%).

**Figure 5:**
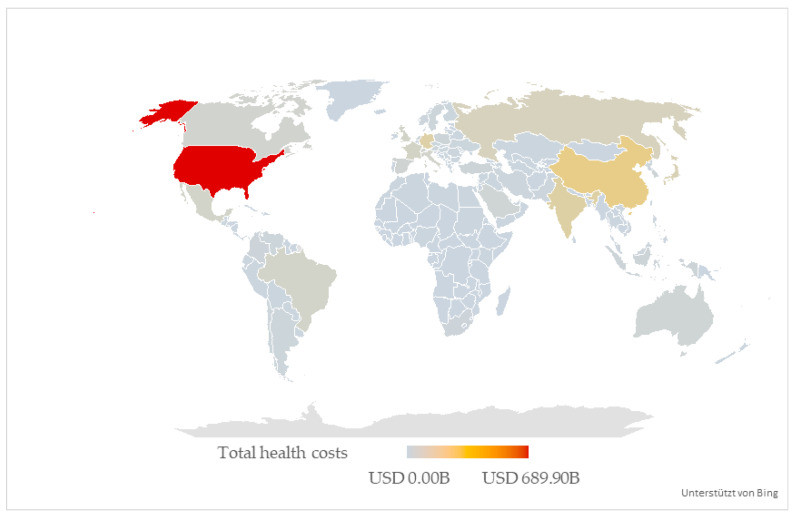
Global distribution of the diet-induced, total health costs for 2024.

The distribution of absolute diet-induced health costs on a global scale is illustrated in Figure 5. A lighter, bluer color indicates a lower share of the global economic burden of nutritional risk factors, while a darker, redder color symbolizes the opposite.

A consideration of the individual countries and territories regarding the influence of underconsumption and overconsumption reveals that the USA incurs the greatest costs in both cases. India is followed by China, Germany, and Japan in terms of the economic burden of underconsumption. If, on the other hand, we look at the absolute costs of overconsumption, China, Germany, Japan, and Russia incur the highest costs after the USA. India—which bears the third-highest global economic burden of diseases caused by poor diets and also ranks among the five countries with the highest costs for underconsumption—has only the thirteenth-highest global monetary burden in terms of food overconsumption.

**Figure 6:**
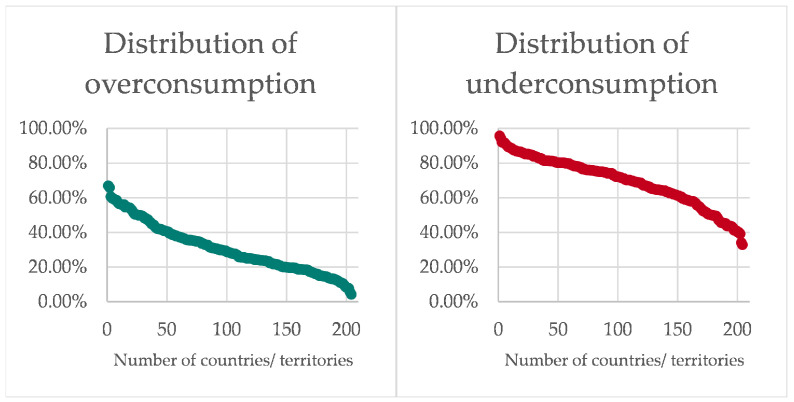
Distribution of country-specific shares of over- and underconsumption of food for 2024.

A comparison of the proportions of deficiently consumed food within countries reveals that Yemen has the highest proportion of underconsumption at 95.79%, followed by Afghanistan with 94.39%, Palestine with 92.14%, DR Congo with 92.08%, and Syria with 91.78%. Overconsumption is particularly prevalent in Brazil; there, 67.07% of the health costs of nutrition are caused by excessively consumed food. Monaco with 65.96%, Andorra with 60.74%, San Marino with 59.82%, and South Korea with 59.68% are also at the top of this ranking. It is evident that underconsumption in certain countries or territories is the predominant cause of the financial implications of malnutrition, while overconsumption has a negligible impact there. A subsequent observation of the corresponding distributions, as shown in Figure 6, across the 204 countries and territories, emphasizes the differing country-specific proportions of the two types of malnutrition.

The average global per-capita health costs of externalities of nutrition are USD 211.08. Of the 204 countries and territories analyzed in our study, 75 exhibit higher costs and 129 lower costs.

The highest per-capita value is in Switzerland with USD 2474.82. This is followed by Monaco with USD 2236.03, the USA with USD 1997.24, Singapore with USD 1887.86, and Iceland with USD 1425.17. The 24 countries and territories with the lowest per-capita health costs due to malnutrition are located exclusively on the African continent.

**Figure 7:**
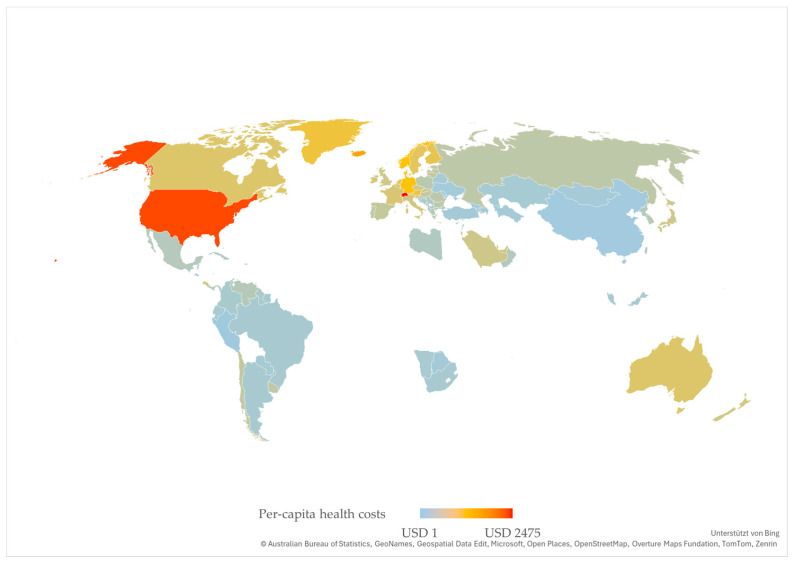
Global distribution of the diet-induced, per- capita health costs for 2024.

Figure 7 shows the level of the per-capita economic burden of the countries and territories considered in our study from a global perspective in that, as with the total costs, lighter, bluer colors represent lower values and darker, redder colors represent higher values.

#### 3.2.2. Comparison of Diet-Induced Health Costs per Continent

Before discussing the diet-induced health costs differentiated by world region, it should be noted that the countries and territories included have been assigned to the continents to which they were also assigned in the GBD study.

A perusal of the data pertaining to the seven continents of the world, with the caveat that information from Antarctica is not available, reveals that North America incurs the highest health costs due to malnutrition. With regard to the financial aspects of the matter, the total for the countries in the aforementioned continent is USD 763.00 billion (44.36% of total costs). The USA accounts for 90.42% of this numerical value, followed by Mexico with 4.50% and Canada with 3.43%. The remaining 24 North American countries and territories thus contribute an aggregate of 1.65% to the malnutrition-related economic burden.

The continent with the second-highest diet-induced health costs is Asia, with USD 420.78 billion (24.46% of total costs). China has the largest share of this amount with 31.06%, followed by India with 20.80% and Japan with 14.84%. The remaining 44 countries and territories in Asia account for 33.30% of the economic burden of diet-induced diseases.

North America and Asia are followed by Europe with USD 407.16 billion (23.67% of total costs). The largest share of this sum is caused by Germany with 20.83%. It is succeeded by Russia with 12.70% and France with 9.97%. In Europe, the remaining 41 countries and territories thus account for the majority of the health costs of nutrition (56.50%).

The fourth-highest costs are incurred in South America at USD 74.98 billion (4.36% of total costs). The most influential countries in this regard are Brazil with a 45.41% share, Colombia with a 11.72% contribution, and Venezuela with a 10.56% proportion. The nine other South American countries are therefore responsible for the remaining 32.31% of health costs for nutrition there.

South America is followed by Africa with USD 32.97 billion (1.92% of health costs). It is evident that the countries exerting the most significant influence are South Africa, with a 30.62% share, Egypt, with a 11.38% contribution, and Algeria, with a 9.34% proportion. The 51 remaining African countries and territories thus account for just under half (48.66%) of the monetized external health effects of nutrition.

The continent with the lowest health costs attributable to over- or underconsumption of food for which data are available is Oceania, with USD 21.04 billion (1.22% of total costs). Australia is primarily responsible for this monetary value, with a share of 83.63%. New Zealand ranks second with a 13.59% share, and Papua New Guinea third with a 1.39% share. The 17 other countries and territories on this continent generate a relative economic burden of 1.39%.

As presented earlier, from a global perspective, slightly more diet-induced health costs are caused by underconsumption than by overconsumption.

The distribution in this regard is heterogeneous across the individual continents. North America is the region in which the share of the economic burden of external health effects of nutrition due to overconsumption of food is highest (55.37% of the costs there).

In South America and Oceania, the cost distributions resulting from both forms of malnutrition are approximately equal. In the former continent, the economic burden of underconsumption corresponds to 49.10%, and that of overconsumption to 50.90%. In Oceania, 49.89% of the diet-induced health costs are attributable to underconsumption of food, while 50.11% are caused by overconsumption.

In Europe and Asia, the majority of people consume insufficient amounts of food. In the former continent, 53.50% of the monetarized health effects of nutrition are caused by underconsumption and 46.50% by overconsumption. In Asia, these proportions are even more skewed; there, 62.97% of the costs are due to underconsumption of food, while 37.03% are due to overconsumption.

In Africa, 73.66% of the diet-induced health costs result from underconsumption and 26.34% from overconsumption. Consequently, it is the continent where the most relative costs are caused due to underconsumption.

When comparing the countries and territories, it was already worked out that the average diet-induced health costs of USD 211.08 per capita are incurred globally. The subsequent step involves the comparison of the mean values of the individual continents.

North America has been identified as the region with the highest per-capita costs. These amount to USD 1247.05 per capita, which is almost six times the amount of the average global monetary burden. This is followed by Europe, with USD 489.47 per capita.

Oceania has the third-highest per-capita health costs at USD 458.32 per capita, which is also above the average. It is followed by South America with USD 172.26 per capita and Asia with USD 89.34 per capita.

The average diet-induced health costs in Africa amount to USD 21.79 per capita, representing 10.32% of the global average.

All aforementioned values are listed in Table 4.

#### 3.2.3. Comparison of Dietary Risk Factors

**Figure 8:**
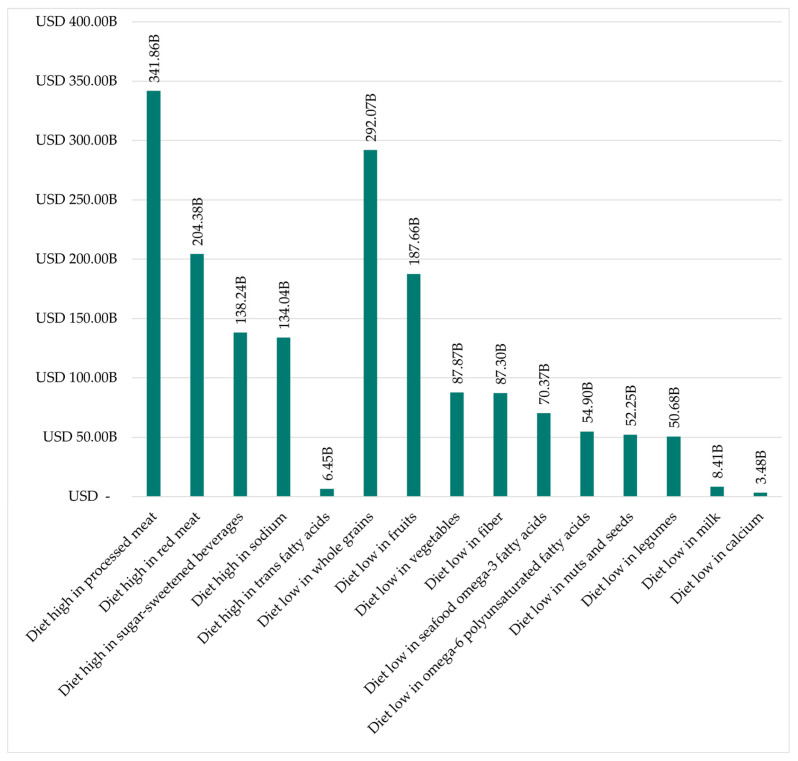
Total diet-induced health costs per dietary risk factor for 2024.

Figure 8 illustrates that the most influential dietary risk factor in 2024 in terms of resulting health costs is the excessive consumption of processed meat at USD 341.86 billion (19.88% of total costs). This is succeeded by diets with a deficient intake of whole grains at USD 292.07 billion (16.98% of total costs) and excessive consumption of red meat at USD 204.38 billion (11.88% of total costs). The underconsumption of fruit leads to USD 187.66 billion (10.91% of total costs), followed by overconsumption of sweetened beverages at USD 138.26 billion (8.04% of total costs).

#### 3.2.4. Comparison of Diet-Induced Health Costs with GDP

**Figure 9:**
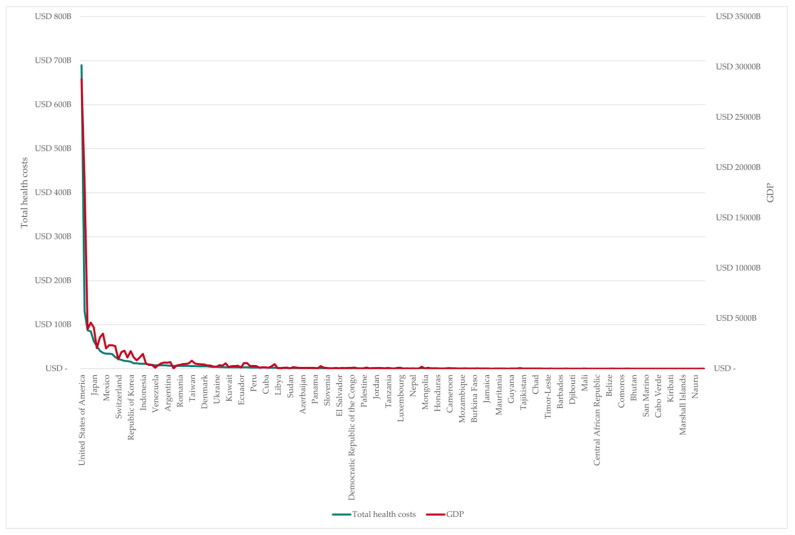
Diet-induced, total health costs, and GDP per country and territory for 2024.

Figure 9 shows the positive correlation between diet-related health costs and GDP in individual countries and territories. This indicates that greater prosperity and wealth lead to higher costs due to poor nutrition.

## 4. Discussion

Following the assessment of the economic burden of CVD, DM, and neoplasms across 204 countries as well as territories and the role of dietary risk factors, this section discusses the results. Thereafter, the possibility of implementing the second SDG, zero hunger, and advancing sustainable nutrition arising from the monetization of externalities will be evaluated.

Supplementary Materials File S1/Section S5 validates the global health costs of the three diseases considered in our study against the background of further scientifically substantiated findings [90,91,92,93]. The results calculated in our study are compared with the results of comparable studies.

Across all three diseases, DALYs and COIs exhibit only moderate correlations, indicating that higher disease burden tends to be associated with greater health expenditures; however, notable anomalies, most prominently the disproportionately high costs in the USA despite comparatively lower DALYs, demonstrate that this relationship is not uniform. In contrast, the correlation between countries’ health costs and GDP is substantially stronger for CVDs (r = 0.89), DM (r = 0.97), and neoplasms (r = 0.97), with far fewer outliers. Consequently, economic prosperity appears to be a much more consistent predictor of health expenditures than disease burden alone. Detailed explanations and descriptions regarding this can be found in Supplementary Materials File S1/Section S5. The accompanying illustrations can be found in Supplementary Materials File S2.

A more pronounced conclusion emerges when the distribution of per-capita health costs for CVDs, DM, and neoplasms is considered. It is apparent that, from a global perspective, there are differences in the healthcare systems and productivity of the individual countries and territories. When taking into account the example of the COI of DM in India and the USA, mentioned above, we can see that a year of life lost in the USA leads to significantly higher costs than in India. It is evident that a DM disease results in increased treatment costs, medication expenses, and other associated expenditures in the USA, which far surpass those observed in India. However, it is equally important to note that the productivity losses incurred as a result of this condition are also more substantial in the USA. When these considerations are expanded to encompass the OPHW explained in the methodology for the comparison and the subsequent estimation of missing indirect cost values, the insight just gained is further emphasized. While the PPP-adjusted OPHW in India in 2024 is INT$ 8.41, this amounts to INT$ 71.13 in the USA [35]. This phenomenon elucidates the occasionally substantial discrepancy observed between the burden of disease and the level of indirect costs, which was initially presumed to be incongruent. Consequently, if an individual were to reduce their working hours by one hour due to the presence of DM in the USA, they would experience a productivity loss that is 8.46 times higher than that of an individual working in India. With regard to direct costs, the accessibility and availability of healthcare facilities [82] (pp. 395–403) and the progressiveness of the healthcare system [94] have a significant influence on costs. In the event of contracting a disease, the loss of life expectancy is inevitable, irrespective of the aforementioned factors. However, in the absence of a hospital or physician in a given country, and, consequently, the inability to obtain a diagnosis, treatment, or medication, no direct costs will be incurred. In another country, where accessibility and availability are guaranteed, these costs will, on the other hand, be experienced. Consequently, the two countries under consideration, which are used as exemplars, will exhibit divergent cost levels for an equivalent disease burden. This represents the main reason why African countries and territories have comparatively low costs with a comparatively high burden of disease.

A higher correlation exists between COI and GDP than between COI and DALYs. This observation indicates that an increase in prosperity may result in enhanced monetized productivity, consequently leading to elevated indirect costs. Moreover, it suggests that the development of healthcare infrastructures and systems can be enhanced with higher prosperity, resulting in higher direct costs for a given disease burden than in another country with less prosperity.

In conclusion, with regard to the health costs of the three diseases CVDs, DM, and neoplasms, it can be stated that there are globally differing ratios between the burden of disease and actual health costs.

Of the total USD 4794.33 billion in health costs attributable to CVDs, DM, and neoplasms, more than one-third, USD 1719.94 billion (35.87%), is due to nutrition. A comparison of this number with USD 1300.00 billion for 2030, mentioned in the Section 1 [28], demonstrates that the value calculated in our study is significantly higher. However, when contextualized against the backdrop of the FAO’s reported external costs amounting to INT$ 8100.00 billion [29], the numerical value appears to be considerably understated, even knowing the underlying currencies are not identical. The disparate outcomes of the aforementioned studies further underscore the significant uncertainty surrounding the precise economic implications of external effects arising from food consumption. As their focus is not exclusively on the determination of health costs, but also on the explanation of other important factors, more general estimation methods are used to calculate these costs. Our study is the first to determine country-specific health costs on a global scale. The general costs of CVDs, DM, and neoplasms were already validated at the beginning of this section by referencing other scientific studies. The plausibility of converting these costs to nutrition using DALYs has previously been established. In summary, it can be stated that the level of health costs in our study is plausible and comprehensible against the background of all the factors just described.

From a global standpoint, the overconsumption (47.96% of diet-induced health costs) and underconsumption (52.04% of diet-induced health costs) of food incur roughly equal health costs. Brazil has the highest proportion of health costs due to overconsumption at 67.07%, while Yemen has the highest relative economic burden due to underconsumption at 95.79%.

It is evident that in each country and territory, a minimum of 32.93% of the financial responsibility for the external health implications of nutrition is attributable to underconsumption, while the proportion of expenditures stemming from overconsumption starts at 4.21%. This leads to the conclusion that deficiently consumed foods and their corresponding risk factors contribute significantly to the health costs of nutrition in every country and territory, whereas this is not the case for excessively consumed foods. This phenomenon can be demonstrated through the utilization of the medians of the two distributions. Across the 204 countries and territories analyzed, the median percentage share for underconsumption is 71.67%, while for overconsumption it is 28.33%.

Considering the distributions analyzed above, taking into account the global share of overconsumption and underconsumption, the question arises as to why both forms of malnutrition generate approximately the same costs despite their different presence in countries and territories worldwide. The underlying reason for this can once again be derived from the different levels of prosperity. As previously shown in the Results Section 3, there is a positive correlation between the level of diet-induced health costs and GDP, which relates greater prosperity to a heavier monetary burden. Among the four countries with the highest financial burden, the USA, China, India, and Germany, we can see that all countries but India incur these costs as a result of overconsumption of food. It is evident that among the 102 countries and territories with the lowest GDPs, only six exhibit higher costs from excessive food consumption compared to insufficient consumption. Of the top half, 20 countries and territories have higher costs due to overconsumption of food than underconsumption. In total, 76.92% of the overconsuming countries and territories analyzed are in the top half of the GDP ranking. Based on this finding and taking into account the positive correlation between GDP and health costs, the different distribution of types of malnutrition can be explained. Countries and territories with greater prosperity tend to consume too much food, which induces illness, while those with less prosperity also consume less food, and the likelihood of illness occurs as a result of this nutritional deficiency. At the same time, countries and territories with higher GDP also tend to incur more costs than those with lower GDP, which means that the overconsumption there is weighted more heavily than the underconsumption in the correspondingly poorer ones. Consequently, from a global standpoint, the economic burden of both types of malnutrition is approximately equivalent, although in a mere 26 of the 204 countries and territories, overconsumption exceeds underconsumption.

When utilizing per-capita health costs as a metric for substantiating this observation, it becomes evident that countries and territories with elevated per-capita GDPs also demonstrate a propensity for higher per-capita health costs. This correlation can be understood in more detail by looking at the corresponding illustration in the ”Supplementary Figures” table in Supplementary Materials File S2.

With regard to cost distribution across continents, it is notable that North America is the only region where the overconsumption rate is significantly higher than the underconsumption rate. The USA accounts for the majority of this burden, representing 90.42% of the continent’s costs. On the other hand, almost three-quarters of the costs in Africa are due to underconsumption. However, while 44.36% of total global diet-induced health costs come from North America, only 1.92% are incurred in Africa. This illustrates once again the previously obtained insight into the unequal weighting of overconsumption and underconsumption.

The analysis of dietary risk factors reveals the significant impact of the USA on the global equal distribution of both overconsumption and underconsumption. The USA ranks first for four of the five risk factors of overconsumption and seven of the ten factors of underconsumption. With regard to the financial implications of the second-placed nation in each instance, it is evident that the USA is at the forefront, particularly with respect to the overconsumption of food. For example, it accounts for over 50% of the costs of overconsumption of processed meat, the biggest risk factor. This already demonstrates how much money a single country can lose due to poor diets.

To summarize, it can be stated that malnutrition leads to more than one-third of the costs of CVDs, DM, and neoplasms. On a global scale, the financial implications of overconsumption and underconsumption of food are approximately equivalent, although the country-specific risk of disease can exhibit significant variation. Countries and territories with a high level of wealth and prosperity are the main contributors to the costs, with overconsumption being the core cause. Conversely, in 178 countries and territories, there is a tendency to consume insufficient rather than excessive amounts. In light of these findings and the fine-grained differentiation of the health costs of nutrition calculated in our study, country-specific recommendations can be developed to address this problem.

The subsequent section will discuss ways in which sustainable nutrition can be promoted using the findings from this study and how these can help to fulfil the goals of the global community, in particular, the second SDG of the global elimination of hunger.

As previously stated in the Section 1 of this study, the principle of sustainable nutrition postulates the promotion of all facets of human health and well-being with a low impact on the environment. In addition to the ecological dimension, the health, social, and economic dimensions also play a role. The results of our study contribute above all to the fulfilment of the health component by showing which diseases occur due to which dietary risk factors in the individual countries and territories, and what costs are incurred as a result. This allows country-specific recommendations to be derived that can help to reduce costs and the incidence of disease. This promotes the prevention of diseases according to the health dimension, which can also increase people’s well-being.

Furthermore, the country-specific internalization of health costs in food prices could lead to fair prices and, thus, to social justice by the social dimension of sustainable nutrition. To date, diet-induced health costs correspond to externalities that distort food prices and therefore do not reflect true prices. Consequently, there is an absence of fair or equitable consumption incentives, which is conducive to the promotion of unhealthy and potentially unsustainable diets.

The country-specific findings of this study provide a robust basis for deriving targeted policy recommendations. As the COI estimates are strongly correlated with prosperity levels, the largest share of monetized diet-related health costs arises in high-income regions, particularly North America and Asia, which account for 44.36% and 24.46% of total global diet-induced health costs, respectively (cf. Table 4). Consequently, political interventions in these regions offer the greatest potential for reducing absolute health expenditures. Due to the high monetized value of productivity losses and healthcare spending in affluent economies, the prevention of a single DALY in these contexts is associated with particularly large cost savings. From an economic perspective, dietary policy interventions in high-income countries, therefore, yield the highest marginal monetary returns. At the same time, this concentration of costs implies that high-income regions also bear a particular responsibility for implementing policy measures that internalize diet-related externalities and contribute to global welfare improvements.

Nevertheless, the availability of country-specific health cost estimates enables the formulation of differentiated strategies beyond high-income regions. Measures that support a gradual convergence of national dietary patterns toward recommended dietary patterns such as the planetary health diet, while remaining socially and culturally viable, have the potential to reduce diet-induced health costs worldwide. Importantly, such measures can be adapted to local disease burdens, consumption patterns, and economic conditions, thereby avoiding one-size-fits-all approaches. This differentiation is crucial from an equity perspective, as identical policy instruments may have markedly different distributional effects depending on income levels, food affordability, and existing dietary constraints across countries and population groups.

Policymakers should prioritize dietary risk factors associated with the highest health costs at the national level. In the case of the United States, for example, diets high in processed and red meat account for 39.66% of total diet-induced health costs. Accordingly, policy efforts should focus on reducing meat consumption, for instance, by increasing prices for meat products, while simultaneously lowering prices for underconsumed food groups such as whole grains (14.30% of total costs) and fruits (9.91% of total costs). One economically efficient approach to reducing external health costs is their partial integration into product prices, thereby increasing the relative prices of unhealthy foods while decreasing those of healthier alternatives. The methodology proposed by Seidel et al. (2023) could be employed to estimate product-specific external health costs [15]. Through demand-side price elasticities, such price signals would be expected to reduce overconsumption of unhealthy foods while encouraging increased consumption of foods currently consumed below recommended levels. Such changes could be achieved through the implementation of fiscal measures such as meat taxes, sugar taxes, or value added tax (VAT) reforms, as well as through non-price-based interventions such as science communication approaches [95].

As shown by Oebel et al. (2024), differentiated VAT rates—which increase prices for overconsumed foods like meat and decrease prices of healthier vegetarian foods—can achieve strong incentive effects while remaining politically and socially implementable [96]. Importantly, such VAT reforms can be designed to be revenue-neutral or even revenue-positive, thereby creating fiscal space that can be used to compensate lower-income households, subsidize healthy staple foods, or support nutrition-sensitive social policies [96].

The WHO recommends fiscal measures such as taxes on sugar-sweetened beverages as a key tool for preventing obesity and diet-related non-communicable diseases, and created a manual [97]. Economically, these taxes are justified as a correction of health costs and behavioral misalignments [98]. Accordingly, numerous countries on all continents have now introduced corresponding instruments. In Latin America, Mexico is considered a pioneer with a national tax since 2014, supplemented by measures in countries such as Chile and Peru. In North America, municipal taxes exist in several US cities (e.g., including Berkeley, Philadelphia, and Seattle). In Europe, prominent examples include the United Kingdom with its Soft Drinks Industry Levy, as well as France, Ireland, Portugal, and Spain (regionally). In Africa, South Africa, with its Health Promotion Levy, and Mauritius, among others, have introduced national sugar taxes. In Asia, corresponding taxes exist in Thailand, the Philippines, Sri Lanka, and parts of India. The empirical evidence on effectiveness is becoming increasingly robust. In Mexico, evaluation studies show that the tax reduced purchases of taxed beverages by around 6% in the first year, by 8–10% in subsequent years, and by as much as 17% in lower-income households, while consumption of untaxed alternatives such as water increased by around 4% [99]. For the United Kingdom, a study on the Soft Drinks Industry Levy shows that the amount of sugar sold in soft drinks fell by around 30%, mainly due to reformulations by manufacturers and less due to pure price effects [100]. Overall, experience from different regions of the world proves that sugar taxes can have a measurable impact on both the supply behavior of industry and the consumption behavior of consumers.

By implementing these measures, high-income countries can set an example and help generate global fiscal space for health-promoting interventions in lower-income regions. Complementary policy instruments include the restructuring of agricultural subsidies, whereby the production of healthier foods is more strongly supported, while subsidies for overconsumed and health-detrimental food products are reduced. Redirecting subsidies in this way can improve access to healthy foods without increasing consumer prices and thus contributes to distributional fairness. Complementary to fiscal reforms, non-price-based interventions such as science communication campaigns, targeted advertising, and nudging can promote behavioral change and support dietary shifts without directly affecting food prices [95].

A full and immediate internalization of external health costs into market prices would likely raise concerns regarding social equity and consumer acceptance, as it could lead to substantial price changes. In particular, lower-income households spend a higher share of their income on food and would therefore be disproportionately affected by abrupt price increases, even if these reflect true societal costs. This underscores the necessity of gradual implementation pathways, complementary policy instruments, and compensatory mechanisms to ensure social acceptability.

A combined policy approach that simultaneously lowers the prices of healthy foods and increases the prices of unhealthy foods is likely to be more effective than isolated measures such as sugar taxes alone. By addressing multiple unhealthy dietary behaviors concurrently, such policy bundles can reduce diet-induced health costs without increasing average household food expenditures. This characteristic is essential for avoiding regressive distributional effects and for ensuring that nutrition-related fiscal policies are perceived as socially just, thereby increasing their political feasibility and long-term effectiveness.

The comprehensive documentation and pricing of the hitherto external health costs of food serve to advance the objectives of the global community. It is hypothesized that fair prices, especially for underconsumed food in poor regions, can potentially lead to a reduction in poverty. The reason for this is the increasing affordability of healthy food, which is essential for life. The third SDG, namely the assurance of health and well-being, could be promoted as described above, while at the same time, sustainable production and consumption in the interests of the environment and humanity could be ensured. International partnerships are also required to facilitate the internalization of health costs on a global scale.

In addition to the goal of the global community described above, the second SDG is also particularly relevant, but has been deliberately omitted as it will be discussed in detail below. As asserted by Braun et al. (2023), in order to achieve the complete eradication of global hunger, an annual investment of USD 265 billion would be imperative [24]. If we compare this number with the aggregated global diet-induced health costs of USD 1719.94 billion, it becomes evident that the global hunger issue could be effectively and efficiently addressed by redistributing these costs. Looking at the costs of excessive consumption of processed and red meat, sodium, and sweetened beverages in the USA alone — totaling USD 387.33 billion — the potential for ending or at least reducing global hunger becomes apparent. In particular, there is an opportunity to capture and redistribute savings from the direct costs of disease.

## 5. Conclusions

At the beginning of our study, we outlined the importance of sustainable nutrition for achieving the SDGs. With regard to nutrition, health-related externalities pose the greatest threat to these goals, yet the majority of studies focus on environmental factors.

The methodology developed in this study, to determine the monetary burden of external health effects across 204 countries and territories, consists of three parts. In the first part, the COI of CVDs, DM, and neoplasms was determined, with heterogeneous or missing data necessitating several intermediate steps. In the second part, the relative burden of these three diseases was calculated based on malnutrition. In the final step, the diet-induced health costs were determined using this information.

Our calculations show that poor nutrition results in annual health costs of USD 1719.94 trillion, representing more than one-third of the total cost associated with CVDs, DM, and neoplasms. The USA accounts for a large share of this, with USD 689.90 billion (40.11% of total global costs), which also means that North America as a whole contributes 44.36% of the monetary burden of malnutrition, while Oceania accounts for only 1.22%. From a global perspective, this translates to USD 211.08 per capita. North America, Europe, and Oceania have above-average per-capita costs, while South America, Asia, and Africa contribute below the global average.

Our global analysis revealed that the financial implications of underconsumption and overconsumption are approximately equivalent. However, at the country level, the economic burden of underconsumption was found to be generally higher than that of overconsumption. Among the continents, North America has the highest relative share of overconsumption at 55.37% of the health costs, while Africa is the region with the highest underconsumption at 73.66%. We observed a highly heterogeneous distribution of costs across all countries and territories, with high-income countries exerting a disproportionate influence on the global economic burden of malnutrition. In particular, the USA accounts for the largest share of diet-induced health costs (USD 689.90 billion), followed by China, India, Germany, and Japan. Together, these five countries account for more than 60% of global costs, while the remaining 199 countries and territories contribute less than 40%. North America as a whole accounts for 44.36% of the monetary burden of malnutrition, while Oceania accounts for only 1.22%. This trend was also confirmed on a continental level. Globally, overconsumption of processed and red meat as well as sweetened beverages is associated with the highest diet-induced health costs. By contrast, whole grain products, fruit, and vegetables in particular are insufficiently consumed. If the external health costs of nutrition were internalized into food prices in a holistic and globally standardized way, the savings could be redistributed in such a way that global hunger could be effectively and efficiently combated, while numerous other SDGs, e.g., the second and third SDGs, could be advanced. The findings of our study demonstrate that global dietary guidelines are as relevant as country-specific recommendations and incentive structures in steering consumption patterns. Both fiscal and non-fiscal policy instruments—such as tax system reforms or nudging strategies to increase fruit and vegetable intake—can effectively support a shift towards diets that are both healthier and more sustainable.

In conclusion, this study answers the research question posed at the outset. Utilizing the method we developed and the methodology of Seidel et al. (2023) [15], we were able to ascertain the diet-induced health costs for 204 countries and territories and derive recommendations for a more sustainable and healthier diet. Our study is the first to quantify the global economic burden of malnutrition at the country level and, thus, provides a highly valuable database for future health research. The findings of this study indicate the necessity for the definition of both country-specific and global measures to incorporate external health costs into food prices, thereby enabling more accurate and equitable food pricing. In addition, it is essential to integrate ecological, social, and economic dimensions when quantifying external costs to ensure the attainment of sustainable nutrition.

## Figures and Tables

**Table 1:** Overview of methods and concepts for quantifying and monetizing health and internalizing externalities.

Study	Methodological Approach	Main Research Topics
[16]	TCA	Explanation of True Cost Accounting (TCA)
[17] (pp. 327–337)	COI	Explanation of cost-of-illness studies (COI): Concepts, methods and differentiation of individual cost types (direct, indirect, intangible)
[18]	GBD data	Statistics on the burden of diseases: classified by disease, continent, country and more

**Table 2:** Overview of studies on (un)healthy and (non)sustainable nutrition, its costs, and the monetary expenditure required to eliminate global hunger.

Study	Geographical Scope	Methodological Approach	Externalities Examined	Main Research Topics/Conclusions
[27]	Global	Food prices and FAO Food Price Index	n.a.	Food safety and the cost of healthy eating by continent and income; 3.96 Purchasing Power Parity (PPP) dollars on a global scale for a nutritionally balanced diet
[24]	Global	Retail prices and Gross National Income and Partial-equilibrium model (Global Agriculture Perspectives System)	n.a.	- Cost of a nutritionally adequate diet: USD 0.71 to USD 1.77 - Cost of eliminating global hunger: USD 265 billion per year
[28]	Global	GBD and COI data as well as connectors from meta-analyses of prospective cohort studies	Environmental and health	Calculation of the global environmental and health costs of food in 2030: - Health costs: approx. USD 1.30 trillion - Environmental costs: approx. USD 1.70 trillion
[29]	Global	TCA	Environmental, social and health	Transformation of agricultural and food systems; Estimation of the hidden costs of food: - Total hidden costs: USD 11.60 trillion - Health costs: USD 8.10 trillion
[15]	Country-specific	TCA, GBD and COI data	Health	Methodology for calculating diet-induced health costs in Germany for 2022: - Total health costs: EUR 50.38 billion - Per-capita health costs: EUR 601.48

**Table 3:** Absolute and per- capita health costs of the countries and territories with the highest populations sizes in 2024.

Country/Territory	Health Costs	Per-Capita Health Costs	Population
India	USD 170.93 B	USD 117.81	1450.94 M
China	USD 513.35 B	USD 361.39	1419.32 M
United States of America	USD 1722.12 B	USD 4985.50	345.43 M
Indonesia	USD 35.69 B	USD 125.89	283.49 M
Pakistan	USD 11.15 B	USD 44.36	251.27 M
Nigeria	USD 4.44 B	USD 19.10	232.68 M
Brazil	USD 116.01 B	USD 547.21	212.00 M
Bangladesh	USD 5.76 B	USD 33.18	173.56 M
Russian Federation	USD 121.29 B	USD 837.53	144.82 M
Ethiopia	USD 1.34 B	USD 10.14	132.06 M

**Table 4:** Diet-induced health costs per continent for 2024: total, differentiated by underconsumption and overconsumption, and per capita.

Continent	Sum of Underconsumption	Sum of Overconsumption	Total Diet-Induced Health Costs	Average Per-Capita Health Costs
Africa	USD 24.28 B	USD 8.69 B	USD 32.97 B	USD 21.79
Asia	USD 264.98 B	USD 155.80 B	USD 420.78 B	USD 89.34
Europe	USD 217.84 B	USD 189.32 B	USD 407.16 B	USD 489.47
North America	USD 340.56 B	USD 422.44 B	USD 763.00 B	USD 1247.05
Oceania	USD 10.50 B	USD 10.54 B	USD 21.04 B	USD 458.32
South America	USD 36.82 B	USD 38.17 B	USD 74.98 B	USD 172.26
Total	USD 894.98 B	USD 824.96 B	USD 1719.94 B	USD 211.08

## Data Availability

The data presented in this study are available in the attached MS-Word and MS-Excel files; these can be found under the link mentioned in the Supplementary Materials Section.

## Supplementary Materials

The following supporting information can be downloaded at https://www.mdpi.com/article/10.3390/nu18030426/s1, File S1: Supplementary Text_Seidel et al.; File S2: Data and Calculations_Seidel et al.

## Abbreviations

The following abbreviations are used in this manuscript:

COI	Cost of illness
TCA	True Cost Accounting
GBD	Global Burden of Disease
CVD	Cardiovascular disease
DM	Diabetes mellitus
UN	United Nations
SDG	Sustainable Development Goal
T2DM	Type 2 diabetes mellitus
GHGs	Greenhouse gases
WHO	World Health Organization
FAO	Food and Agriculture Organization of the United Nations
PPP	Purchasing Power Parity
DALY	Disability-adjusted life year
YLD	Years lived with disease
YLL	Years of live lost
CPI	Consumer Price Index
GDP	Gross Domestic Product
INT$	International Dollar
OPHW	Output per hour worked
EU	European Union
A&E	Accident and Emergency
IHD	Ischemic Heart Disease
NCD	Non-communicable disease
HHD	Hypertensive Heart Disease
T1DM	Type 1 diabetes mellitus
ILO	International Labour Organization
