# Supplementary material for: Determining the Global Economic Burden of External Health Effects of Food Consumption in 204 Countries and Territories
Source: Nutrients. 2026 Jan 28;18(3):426. doi: 10.3390/nu18030426 (PMC12900027; doi:10.3390/nu18030426)
Supplement: Supplementary file 1 [file nutrients-18-00426-s001.zip › Supplementary Materials File S1.pdf]

nutrients

MDPI

Supplementary Materials File S1

Determining the global economic burden of external health effects of food consumption in 204 countries and territories – Supplementary Materials File S1

Felix Seidel, Benjamin Oebel, Lennart Stein, Susanne Kleemann and Tobias Gaugler

S1. Method for calculating the COI for partial costs - detailed procedures

The characteristics of the individual cases and possibilities are evaluated and formulated in detail below. The general logic here corresponds entirely to that mentioned in the main text.

The result of all three possibilities with regard to the first case is the total COI of the disease under consideration in the respective country, year and currency, taking into account current disease burden ratios ($C_{x,d,y,cur}$).

The first possibility in the first case, where information on direct ($c_{dir,x,sd,y,cur}$) and indirect costs ($c_{ind,x,sd,y,cur}$) is available, is the relation of these to a sub-disease (sd), i.e. a particular manifestation of the disease under consideration. Here, the current (y2) prevalence of these subgroups in the respective country or territory ($P_{x,sd,y2}$) is determined and set in relation to the overall prevalence of the disease ($P_{x,d,y2}$).

In the event of the presence of both COI proportions, both ratios are standardised by prevalence within the same system.

$$C_{x,d,y,cur} = (c_{dir,x,sd,y,cur} + c_{ind,x,sd,y,cur}) \div \frac{P_{x,sd,y2}}{P_{x,d,y2}} \quad (8)$$

The second possibility of the first case is the availability of data on direct ($c_{dir,x,d,ag,y,cur}$) and indirect costs ($c_{ind,x,d,ag,y,cur}$) of a certain age group (ag) and additionally on the current prevalence of the disease for the age group ($P_{x,d,ag,y2}$) and the corresponding overall prevalence ($P_{x,d,y2}$) of the disease. Furthermore, information is required on the general ratios of the two shares ($s_{dir,x,d}$; $s_{ind,x,d}$) in the total COI. It is important to note that the allocation of direct and indirect costs can be adjusted based on the average of the respective age group. The direct part is to be transferred directly to the population as a whole, while the indirect costs depend on the productivity delivered by one. The former share can be divided by the prevalence for the purpose of extrapolation, while the latter share is based on the general ratios.

$$C_{x,d,y,cur} = (c_{dir,x,d,ag,y,cur} \div \frac{P_{x,d,ag,y2}}{P_{x,d,y2}}) \times (1 + \frac{s_{ind,x,d}}{s_{dir,x,d}}) \quad (9)$$

The third possibility of the first case concerns the existence of data on the direct ($c_{dir,x,sd+nd,pat,pub,y,cur}$ and $c_{dir,x,sd+nd,pat,priv,y,cur}$) and indirect ($c_{ind,x,sd+nd,pat,pub,y,cur}$ and $c_{ind,x,sd+nd,pat,priv,y,cur}$) per-patient costs (pat) of a top category (sd+nd) of the analysed disease in public (pub) or private hospitals (priv). This disease is divided into a subcategory (sd) and a category which is not directly related to the disease under investigation (nd). In order to ascertain the representative current costs, it is necessary to divide the prevalence of the subcategory ($P_{x,sd,y2}$) by the sum of itself and the prevalence of the non-relevant

proportion ($P_{x,nd,y2}$). The relative prevalence of the analysed disease is then calculated, i.e. the proportion of the total number of cases of the subcategory ($P_{x,sd,y2}$) to the total number of cases of the disease ($P_{x,d,y2}$). This is first carried out for the costs incurred in public hospitals.

$$c_{dir,x,d,pat,pub,y,cur} = c_{dir,x,sd+nd,pat,pub,y,cur} \times \frac{P_{x,sd,y2}}{P_{x,sd,y2}+P_{x,nd,y2}} \div \frac{P_{x,sd,y2}}{P_{x,d,y2}} \quad (10)$$

$$c_{ind,x,d,pat,pub,y,cur} = c_{ind,x,sd+nd,pat,pub,y,cur} \times \frac{P_{x,sd,y2}}{P_{x,sd,y2}+P_{x,nd,y2}} \div \frac{P_{x,sd,y2}}{P_{x,d,y2}} \quad (11)$$

The direct and indirect per-patient costs of the disease in question in private healthcare facilities are then calculated according to the same system.

$$c_{dir,x,d,pat,priv,y,cur} = c_{dir,x,sd+nd,pat,priv,y,cur} \times \frac{P_{x,sd,y2}}{P_{x,sd,y2}+P_{x,nd,y2}} \div \frac{P_{x,sd,y2}}{P_{x,d,y2}} \quad (12)$$

$$c_{ind,x,d,pat,priv,y,cur} = c_{ind,x,sd+nd,pat,priv,y,cur} \times \frac{P_{x,sd,y2}}{P_{x,sd,y2}+P_{x,nd,y2}} \div \frac{P_{x,sd,y2}}{P_{x,d,y2}} \quad (13)$$

The resulting values are afterwards combined with the proportions of hospital visits in public ($w_{x,pub}$) or private buildings ($w_{x,priv}$) in the respective country or territory. The direct part is adjusted using the hospitalization rate of the disease in this country or territory ($h_{x,d}$) in order to attribute the costs to the hospital visits that actually took place, and, for example, the medication prescribed as a result. To ascertain the direct COI, it is necessary to take into account the prevalence of the disease in the respective country or territory and year. The indirect cost share requires the prevalence of CVD sufferers who are not retired and working ($P_{x,d,w,y}$). In this study, it is hypothesised that indirect costs are incurred exclusively in the context of employment.

$$C_{x,d,y,cur} = w_{x,pub} \times \left(c_{dir,x,d,pat,pub,y,cur} \times P_{x,d,y} \times h_{x,d} + c_{ind,x,d,pat,pub,y,cur} \times P_{x,d,w,y}\right) + w_{x,priv} \times \left(c_{dir,x,d,pat,priv,y,cur} \times P_{x,d,y} \times h_{x,d} + c_{ind,x,d,pat,priv,y,cur} \times P_{x,d,w,y}\right) \quad (14)$$

In the second case, information is available on one part of the COI (sCOI), so on either direct or indirect costs (c_{sCOI}). The result is always the partial COI of the disease under consideration in the respective country, year and currency, taking into account current disease burden ratios ($c_{sCOI,x,d,y,cur}$).

The first possibility occurs in instances where a cost component ($c_{sCOI,x,sd,y,cur}$) is associated with a specific subcategory of the disease. Here, as in the first possibility of the first case, the corresponding partial COI is determined using the ratio of the current prevalence of the subgroup ($P_{x,sd,y2}$) and the overall prevalence of the disease ($P_{x,d,y2}$).

$$c_{sCOI,x,d,y,cur} = c_{sCOI,x,sd,y,cur} \div \frac{P_{x,sd,y2}}{P_{x,d,y2}} \quad (15)$$

As no age groups are considered in our study with regard to the second possibility of the second case, the equivalent procedure is not discussed in more detail here. In terms of systematics, this corresponds to the second possibility of the first case, whereby the existence of general ratios of direct and indirect costs is essential.

The third and final possibility of this case is that either the direct or the indirect per-patient costs ($c_{sCOI,x,sd,pat,y,cur}$) of a subcategory of the disease are available. In this case, too, the current overall prevalence of the disease in the respective country ($P_{x,d,y2}$) and that of the subgroup ($P_{x,sd,y2}$) are required. The resultant figure is the per-patient cost of the COI share of the disease under consideration in the respective country, year and currency.

$$c_{sCOI,x,d,pat,y,cur} = c_{sCOI,x,sd,pat,y,cur} \div \frac{P_{x,sd,y2}}{P_{x,d,y2}} \quad (16)$$

A distinction must now be made between direct and indirect costs. If direct costs are specified (sCOI = dir), the value calculated is multiplied by the current hospitalization rate ($h_{x,d}$) and the prevalence of the disease in the country in the respective year ($P_{x,d,y}$).

$$c_{sCOI,x,d,y,cur} = c_{sCOI,x,d,pat,y,cur} \times P_{x,d,y} \times h_{x,d} \quad \forall\ sCOI = dir \quad (17)$$

If there are indirect costs (sCOI = ind), the value is multiplied by the prevalence of the country's working population ($P_{x,d,w,y}$) in the corresponding year.

$$c_{sCOI,x,d,y,cur} = c_{sCOI,x,d,pat,y,cur} \times P_{x,d,w,y} \quad \forall\ sCOI = ind \quad (18)$$

The other COI share is determined using the methodology outlined in the following subsection.

The third case occurs if only data on the total COI, i.e. the sum of direct and indirect costs, are available for a subcategory ($C_{x,sd,y,cur}$) and no further information is obtainable on the distribution of both shares. Once again, the current overall prevalence of the disease in the country under consideration ($P_{x,d,y2}$) and that of the subcategory ($P_{x,sd,y2}$) are required. In this case, the total COI of the disease under consideration in the respective country, year and currency is obtained, considering current disease burden ratios ($C_{x,d,y,cur}$).

$$C_{x,d,y,cur} = C_{x,sd,y,cur} \div \frac{P_{x,sd,y2}}{P_{x,d,y2}} \tag{19}$$

The missing COI shares are then determined using the corresponding score-based comparison procedure.

As only subcategories of diseases in the third case are considered in this study, the two other options are not explained further. Nevertheless, the procedure under discussion is analogous to that previously outlined.

S2. Score-based comparison with missing information

The following explanations describe in detail how missing information is handled in the estimation of the two types of health costs using the score-based comparison.

The procedures for estimating missing costs are not applicable in the event that individual data are absent during the calculation of scores and the scale factor. In the following discussion, we will therefore explore methods for estimating these costs.

In both cases, when determining the direct and indirect COI, the four factors DALY-share$_{x,d,y}$, Deaths-share$_{x,d,y}$, I-share$_{x,d,y}$ and P-share$_{x,d,y}$ are used to quantify the respective burden of disease. It is important to note that these data are consistently available for all countries and territories considered in our study.

For the first factors, the proportion of PPP-adjusted per capita healthcare costs to PPP-adjusted per capita GDP and OPHW, corresponding data are absent for several countries and territories. In this case, the emphasis is exclusively on the burden of the disease. The respective score for the ensuing construction of the distance matrix is calculated from 0.3 each of the DALY-, I- and P-shares and 0.1 of the Deaths-share and corresponds to the burden share already discussed.

$$Score_{missing,x,d} = Burden - share_{x,d,y} = 0.3 \times DALY - share_{x,d,y} + 0.1 \times Death - share_{x,d,y} + 0.3 \times I - share_{x,d,y} + 0.3 \times P - share_{x,d,y} \tag{32}$$

The scale factor ($SF_{missing,x,d}$) for the subsequent estimation of the direct COI of the respective country or territory is calculated accordingly from the product of the population size in 2024 and the burden of disease, formalised as the burden share.

$$SF_{missing,x,d} = p_{x,2024} \times Burden - share_{x,d,y} \tag{33}$$

It is imperative to note that the calculation is less precise in the absence of a health cost share. Consequently, the results must be adjusted accordingly. In this case as well, the validity of the resulting estimates is ensured based on countries and territories for which data are available. A pivotal figure is identified in this context, which can be utilized to implement systematic adjustments.

S3. Input data for quantification and monetization

The subsequent section delineates the sources and data utilized to ascertain the economic burden of external health effects of nutrition for 204 countries and territories.

S3.1 General input data

This subchapter provides an overview of the key data required for the analysis and their sources.

Exchange rates for the relevant currencies are primarily derived from the monthly historical data published by the Board of Governors of the Federal Reserve System, from which annual averages are calculated [36]. Since these data are only available from 2015 to 2024 and not for all currencies needed, additional sources are consulted [37-40]. After converting all monetary values into USD, inflation adjustments are applied [41].

In the context of our study, which involves the calculation of costs for entire populations, a concurrent demographic adjustment at the country or territory level is imperative, in addition to the inflationary adjustment. This calculation is derived by applying the respective population sizes from the year of the data source and 2024. All population figures from 1960 to 2023 are extracted from a World Bank database [42]. The data for the year 2024 are drawn from the Worldometer [43], which elaborates on UN information and is based on the forecasts of the world population [44].

All values relating to the burden of disease are taken from the aforementioned GBD Compare tool from the University of Washington [23]. To conduct the score-based comparison, additional data on PPP-adjusted and real per capita health expenditure from 2021 [45] as well as PPP-adjusted per capita GDP from 2023 [46] are also applied. Although corresponding information up to 2023 is available for the latter, it is partially incomplete, and the use of data from 2021 are required for offsetting against the health expenditure values due to the necessary uniformity. The population data and comparative costs are retrieved from the sources already mentioned.

In order to ascertain the reference countries and territories for estimating the indirect COI, it is also necessary to determine the OPHW, which indicates the respective productivity for the year 2024 [47].

A detailed list of all scores, distance matrices, scale factors and the final score-based comparison per disease, broken down by country and territory, can be found in the corresponding tables inSupplementary Materials File S2.

S3.2 Input data for determining the monetary burden of disease

The sources used to determine the COI for CVD, DM and neoplasms are presented below. **Error! Reference source not found.** lists all referenced sources, providing detailed information on the disease examined, the COI part, the form of partiality, the countries or territories investigated and the year of the data.

Table S1. Cost-of-illness data sources, broken down by disease studied, cost component, partiality options, countries or territories studied, and years of the cost data.

Main disease	Authors	Disease(s) investigated	COI component investigated	Partiality?	Countries/ territories investigated	Year(s) of cost data
**CVD**	[48] (pp. 4752-4767)	CVD	Direct and indirect	No	Austria, Belgium, Bulgaria, Croatia, Cyprus, Czech Republic, Denmark, Estonia, Finland, France, Germany, Greece, Hungary, Ireland, Italy, Latvia, Lithuania, Luxembourg, Malta, Netherlands, Poland, Portugal, Romania, Slovakia, Slovenia, Spain, Sweden	2021
	[49]	CVD	Direct and indirect	No	United Kingdom	2015
	[50]	CVD	Direct and indirect	No	Bahrain, Kuwait, Oman, Qatar, Saudi Arabia, UAE	2019
	[51];<br>[52]	CVD	Direct and indirect	No	Japan<br>Brazil	2014<br>2015
	[53]	CVD	Direct	No	USA	2020
	[54]	CVD	Indirect	No	USA	2020
	[55] (pp.349-356);<br>[56] (pp. 12-26)	CVD	Direct and indirect	No	China	2003
	[57]	CVD	Direct and indirect	No	Canada	2004
	[59] (pp. 199-204)	CVD	Direct and indirect	No	Russia	2009
	[60] (pp. 137-143)	CVD	Direct and indirect	No	Serbia	2009
	[61] (pp. 235-240)	CVD	Direct and indirect	No	Turkey	2016
	[62] (pp. 57-66)	CVD	Total	No	Switzerland	2021
	[63]	Ischaemic heart disease, stroke	Direct and indirect	Subdiseases	Taiwan, South Korea, Thailand, Singapore	2016
	[65]	Heart failure, arterial fibrillation, hypertension, myocardial infarction	Direct and indirect	Sub-diseases	Chile, Colombia, Ecuador	2015
	[66] (pp. 1212-1219)	Myocardial infarction, stroke	Direct and indirect	Subdiseases	Australia	2020
	[67] (p. 1355);<br>[68]	CVD	Direct and indirect	Age group	Iran	2021

	[75]; [76]; [77]	Hypertension	Direct and indirect	Sub-disease and per capita costs	Nigeria	2019
	[78]	Cardiac diseases, cerebrovascular diseases	Direct	Subdiseases	Argentina, El Salvador, Trinidad and Tobago	2020
	[79] (p. 140)	Ischaemic heart disease, ischaemic stroke, haemorrhagic stroke, hypertensive heart disease	Direct	Sub-diseases and per patient costs	Cameroon	2017
	[80]	CVD	Direct	Per patient costs	India	2018
	[81]	Heart diseases	Total	Sub-diseases	Mexico	2015
**DM**	[82] (pp. 963-970)	DM	Direct and indirect	No	179 countries and territories	2015
**Neoplasms**	[83] (pp. 465-472)	Neoplasms	Direct and indirect	No	204 countries and territories	2020 - 2050

Looking at the global number of DALYs caused by dietary risk factors, it becomes clear that of the 178.26 million DALYs evoked by dietary risk factors in 2021, a total of 169.73 million (95.22%) were caused by CVD, DM and neoplasms [23]. This underscores the focus on these three diseases described in the introduction with numerical values.

With regard to the subject of CVD, there is an absence of research that determines the COI from a global perspective for all or the majority of countries and territories. However, there is literature addressing this for a group or individual countries. Studies are available for both DM and neoplasms that provide a global overview of the external health costs of nutrition, covering most or all of the countries and territories utilized in the GBD study.

In principle, our research prioritises studies that include multiple countries or territories with the aim of achieving partial uniformity concerning the methodologies employed. In instances where the validity of the estimates may be contested based on comparisons with other sources, there is flexibility to deviate from the established prioritisation through the utilisation of individual studies that employ presumably more adequate estimation methods. Moreover, studies that estimate complete COI data are prioritised.

The following section discusses the individual studies that determine the health costs of CVD for a group or individual countries and territories.

As there is an absence of research that encompasses all or a substantial proportion of the 204 countries and territories contemplated in our study to ascertain the burden of disease of CVD, we principally endeavour to draw on international studies that consider multiple countries and territories. In order to determine the health costs of CVD, it is necessary to combine several studies, as the largest study covering multiple countries and territories includes 27 of the 204 countries and territories contained in our study. However, the procedure is identical throughout. In the first step, the existing costs are determined before being standardised and homogenised in the second step using the methods already explained. Finally, they are converted uniformly into USD at the corresponding exchange rate and extrapolated for both inflation and demographics for the year under consideration. First, the costs of the countries and territories with complete COI are determined, before those with partial data are then calculated. A detailed list with the calculation components and final costs of the individual countries and territories, divided into tables, can be found in Supplementary Materials File S2in the ‘CVD COI’ table.

For the European Union (EU), a study is utilised that differentiates both the direct and indirect costs of CVD in detail. The study covers 27 countries and is the most comprehensive to date in terms of its focus on CVD. With regard to the direct share, a distinction is made between healthcare and social care. The former comprises primary care,

outpatient care, accident and emergency (A&E), hospital care and medications. The second component consists of institutionalisation and home care. The indirect part differentiates between informal care and productivity losses, with the latter again being incurred in the form of mortality and morbidity. The study provides a quantitative analysis of the costs incurred in 2021, expressed in EUR. In total, EUR 155.39 billion in direct costs and EUR 126.15 billion in indirect costs are generated. Germany has the highest share of both direct costs at EUR 44.87 billion (28.87% of the total direct costs of the 27 countries) and indirect costs at EUR 38.54 billion (30.55% of the total indirect costs of the 27 countries). That means that the total cost share is also the highest at EUR 83.41 billion (29.62% of the total costs of the 27 countries). This is followed by Italy with EUR 41.50 billion in direct and indirect costs (14.91% of the total costs of the 27 countries) and France with EUR 38.14 billion EUR (13.55% of the total costs of the 27 countries) [48] (pp. 4752-4767).

The conversion of costs is then undertaken in accordance with the aforementioned uniformity principle, employing the USD-EUR exchange rate of USD 1.18 per EUR for 2021 [36], as previously described. This is subsequently adjusted for inflation using consumer price indices and demographically using population data for the year 2024.

In order to estimate the COI of the United Kingdom, it is necessary to use an older study from the European Heart Journal from 2015. This is attributable to the United Kingdom's exit from the EU. In the aforementioned study, the direct share is comprised of primary care, outpatient care, A&E, inpatient care and medications. The indirect costs consist of productivity losses due to mortality, morbidity and informal care. In 2015, the United Kingdom incurred a total of EUR 26.67 billion in health costs due to CVD, of which EUR 12.35 billion (46.31% of the costs) were due to direct factors and EUR 14.32 billion (53.69% of the costs) were the result of indirect, non-healthcare costs [49]. These figures must then be converted using the exchange rate of USD 1.11 per EUR [36] from 2015, and subsequently adjusted for inflation and demographics to the year 2024.

The second largest study in terms of the total cost of CVD includes data for the six countries of the Gulf Cooperation Council, Bahrain, Kuwait, Oman, Qatar, Saudi Arabia and the United Arab Emirates. A prevalence-based, bottom-up COI approach is utilised to estimate the direct costs, with the process taking into account such factors as salaries, treatment and procedures. The indirect portion of the COI comprises productivity losses due to absence, attendance and premature death. The study calculates the costs already converted into USD for 2019, totalling USD 13.14 billion in direct costs and USD 17.40 billion in indirect costs for these six countries. Saudi Arabia has the highest share of both direct and indirect costs with USD 8.22 billion (62.58% of the direct costs of the six countries) and USD 5.62 billion (32.29% of the indirect costs of the six countries) respectively, resulting in total costs of USD 13.84 billion (45.32% of the total costs of the six countries). This is followed by the United Arab Emirates with USD 7.13 billion in total costs (23.34% of the total costs of the six countries) and Qatar with USD 3.66 billion (11.97% of the total costs of the six countries) [50].

As the study under consideration already states the costs in USD, it is not necessary to perform a subsequent conversion using the exchange rate. Instead, only an inflationary and demographic adjustment to the year 2024 is required.

The last study on CVD, which takes a holistic view of several countries and summarises them with complete COIs, undertakes a cost analysis for a selection of G20 countries. The COI estimates are compiled for the USA, Japan, the United Kingdom, Brazil and Mexico [51]. For the USA, the United Kingdom and Mexico, our study employs corresponding costs from more recent studies or research, which provides a more realistic estimate of the monetary burden. For Brazil and Japan, the total costs are determined for 2015 and 2014, respectively [52]. As defined in the literature, these are comprised of two categories of cost, namely direct healthcare costs and indirect costs, which manifest in the form of

productivity losses and human capital losses. From a more granular perspective, the direct share consists of current healthcare expenditure and personal healthcare. The latter is defined as a combination of care, medical goods and additional services. Collective services, including prevention, public health services and health administration, are also included [51]. The total costs for Japan are USD 109.60 billion, and USD 17.30 billion for Brazil [52]. The direct costs in the former country amount to USD 49.50 billion, whereas the latter country incurs direct costs of USD 10.90 billion. These monetary values are then adjusted for inflation and demographics for the year 2024.

For the USA, a study is used that calculates the COI for 2020. The direct healthcare costs are estimated using the Medical Expenditure Panel Survey [53]. The survey under discussion determines the annual healthcare expenditure, and as such includes both payments made by insurers and out-of-pocket payments made by patients. The indirect costs are constituted by productivity losses resulting from morbidity and premature death. In total, it is estimated that CVD incurred USD 627.00 billion in health costs in 2020. Of these, USD 393.00 billion (62.68% of the costs) correspond to direct costs, while the remaining USD 234.00 billion (37.32% of the costs) represent the indirect share [54]. As these costs are already stated in the correct currency, they only need to be adjusted for inflation and demographics for the year 2024.

A combination of two studies is used for China. The first estimate calculates the direct costs for 2003 at USD 26.10 billion [55] (pp. 349-356). This study employs a prevalence-based COI approach. The direct costs consist of inpatient costs, outpatient visit costs, self-treatment and other treatments. The second component is defined as the direct nonmedical costs, which consist of transport, nutrition and caretaker. However, as this study does not provide any information on the indirect share, a second study is required to determine the corresponding percentages. In this instance, the proportion of direct costs is estimated at 51.09% and the proportion of indirect costs at 48.91% [56] (pp. 12-26). Utilising these ratios, the indirect and total COI value can be calculated by multiplying the respective shares by the direct costs of the first study. This results in USD 24.98 billion in indirect costs and USD 51.08 billion in total costs for 2003. These figures are then adjusted for 2024 using the corresponding inflation and population data.

For Canada, a study is utilised that estimates the COI for 2004 in CAN$. A total of CAN$ 21.2 billion was incurred, of which CAN$ 7.84 billion (36.98% of the costs) were due to direct factors and CAN$ 13.36 billion (63.02% of the costs) due to indirect factors [57]. The most significant direct cost factor is hospitalization, followed by drugs and physician care. In the indirect portion, premature death is the main contributor, followed by long-term disability and short-term disability [58].

In order to ascertain the Russian CVD COI, data are retrieved from a study that estimates the corresponding values for the years 2006 to 2009 in EUR and roubles. In our study, the most recent data available is utilised, thus encompassing those from 2009. A distinction is made between direct health care costs and indirect costs, which are referred to in this study as costs outside the health care sector. The financial implications of healthcare services encompass a wide range of costs, including primary and outpatient care, A&E, inpatient care, cardiosurgery and percutaneous coronary interventions as well as medication. The indirect costs include productivity losses due to premature death and morbidity, as well as pensions for disabilities. It is evident that informal care and out-of-pocket payments were not included in the study due to the paucity of available information. In total, EUR 24.40 billion in costs were incurred in Russia in 2009 due to CVD diseases. The majority of these costs can be attributed to indirect payers, which totalled EUR 19.20 billion (78.67% of the costs), while direct costs had a smaller impact at EUR 5.20 billion (21.33% of the costs) [59] (pp. 199-204). As the values are given in EUR, they must

be converted to USD using the exchange rate of USD 1.39 per EUR in 2009 [40] and then adjusted for inflation and population change for the year 2024.

To determine the Serbian health costs of CVD, a study is chosen that follows a top-down approach. The underlying system is defined as the aggregation of data pertaining to both direct and indirect costs. The former refers to healthcare costs, which consist of hospitalization, surgical and diagnostic procedures experienced by the respective patient. Medication, visits to physicians at primary care and rehabilitation are also included. The indirect costs are estimated in this study using a human capital approach, taking into account the lost income due to mortality and morbidity, i.e. productivity losses. In 2009, a total of EUR 514.28 million in health costs were incurred in Serbia due to CVD. The majority of the costs of EUR 400.35 million (77.85% of the costs) are direct cost drivers, while EUR 113.92 million (22.15% of the costs) are indirect costs [60] (pp. 137-143). As the values are stated in EUR, they must be converted into USD using the 2009 USD-EUR exchange rate, which was previously utilised in the Russian case at USD 1.39 per EUR [40], prior to extrapolation for 2024 in inflationary and demographic terms.

The COI of CVD in Turkey is determined by an additive burden of illness model, which estimates the direct and indirect health costs for 2016. The direct share is defined as the proportion of the costs directly attributable to the disease. In this study, these correspond to the costs of recognising, treating and rehabilitating CVD. The indirect costs are defined as the loss of income attributable to the disease. In this context, the aforementioned costs encompass premature death, early retirement and hospitalization. The human capital approach, as previously referenced, is utilised for the calculation. The COI of CVD in Turkey in 2016 is found to amount to USD 10.2 billion, of which USD 3.40 billion corresponded to direct costs (33.33% of the costs) and USD 6.80 billion to indirect costs (66.67% of the costs) [61] (pp. 235-240). The amounts are already stated in USD, which is why they are only adjusted for inflation and population data for 2024.

The last country for which data on the total COI of CVD is available is Switzerland. The study determines the sum of direct and indirect costs for the year 2021. These amounted to CHF 27.80 billion, with the sum of socio-economic costs corresponding to 4% of the GDP [62] (pp. 57-66). The costs are then standardised using the exchange rate of USD 1.09 per CHF in 2021 [36] and subsequently adjusted for inflation and demographics to the year 2024.

The data sources for countries where only partial costs are available are given below. The first countries and territories under consideration are those for which data on direct and indirect costs are available. However, these data relate to either only a subcategory of CVD, to a specific proportion of the population or a combination of an age group, a top category of CVD and the differentiation of the sector.

The third largest study in terms of the number of countries included, examines the direct and indirect costs of countries in Asia. The data for Taiwan, South Korea, Thailand and Singapore are included in our study, while those for China, Australia and Japan are derived from other studies. The rationale behind the exclusion of China pertains to the ambiguity surrounding the designated national territory and a possible underestimation of costs. More recent studies have been conducted for Australia and Japan, which provide a more accurate estimate of the costs by assumption. The cost level in Singapore can be validated by means of a further study [63], while no studies with comparable information content to the one used can be identified for Taiwan, South Korea and Thailand. In the case of the four aforementioned countries, the direct costs appear as a result of diagnosis and medical treatment are described as healthcare costs. A distinction can be made between hospitalization, drugs, rehabilitation and outpatient care. The indirect share comprises productivity losses arising from absenteeism and early retirement, as well as tax losses. The costs of informal care and premature death are also encompassed in this

analysis. This study employs a prevalence-based COI approach, which estimates the costs of the individual countries for 2016. A differentiation is drawn between ischaemic heart disease (IHD) and stroke, the sum of which gives the total economic cost of CVD as defined in this study [64].

The values are then extrapolated to the entirety of CVD according to the first possibility of the first case, using the current ratio of the overall prevalence of CVD and the prevalence of the subcategories, in this case, IHD and stroke. Following adjustment for inflation and demographic factors, the direct costs incurred by the selected four countries in 2024 amount to USD 24.88 billion (43.78% of the costs) and the indirect costs to USD 31.95 billion (56.22% of the costs). The highest costs are experienced in South Korea, totalling USD 22.10 billion in total costs (38.88% of the costs), followed by Singapore with USD 21.53 billion (37.88% of the costs) and Taiwan with USD 9.83 billion (17.29% of the costs). Thailand has the lowest CVD COI in this ranking with USD 3.38 billion (5.94% of the costs). The total percentage shares correspond to both the direct and indirect shares, which are equivalent in amount.

For Chile, Colombia and Ecuador, a study is utilised that analyses the economic burden of heart disease in the respective countries. It equals the first possibility of the first described case of the method for calculating the COI for partially existing costs. A prevalence-based approach is used for heart failure, arterial fibrillation and hypertension, while an incidence-based approach is employed for myocardial infarction. All of these diseases mentioned above represent subcategories of heart disease. The direct costs are equivalent to the healthcare system costs resulting from healthcare expenditure. The indirect share comprises productivity costs due to absenteeism or mortality, the opportunity costs associated with informal care and tax losses of the people with illness and their carers. The study calculates the costs for 2015 for Chile in Chilean pesos, for Colombia in Colombian pesos and for Ecuador in USD [65]. As with cerebrovascular and cardiac diseases, heart disease is classified as a subcategory of CVD. Consequently, an additional extrapolation is necessary, utilising the prevalence data from the GBD Compare tool. The corresponding share of the subcategories in the total number of CVD cases is 38.50% for Chile, 51.33% for Colombia and 65.39% for Ecuador [23]. For the first two countries, it is necessary to convert the resulting COI values from pesos to USD. In 2015, the exchange rate of Chilean pesos to USD was 0.0015, while the rate for Colombian pesos was 0.0004. All costs in USD will subsequently be subjected to inflation and demographic adjustments for the year 2024. The total COI for these three countries is thus USD 24.86 billion, of which USD 9.38 billion (37.71% of the costs) are direct costs and USD 15.48 billion (62.29% of the costs) are indirect costs. Chile has the highest total costs at USD 10.80 billion (43.45% of the costs), followed by Colombia with USD 10.77 billion (43.33% of the costs) and Ecuador with USD 3.29 billion (13.22% of the costs).

In order to ascertain the economic burden of CVD in Australia, a study is conducted that estimates the health and economic impact of this disease between 2020 and 2029. A two-stage multistate dynamic model is employed with the limitation of CVD to myocardial infarction and stroke. Consequently, an adjustment via prevalence rates is necessary. The direct healthcare costs and the indirect costs resulting from productivity losses are taken into account. The latter are determined by reduced salaries due to absenteeism, retirement and monetary losses due to premature death. The resulting costs are also listed separately by gender as well as by primary or secondary prevention and are always stated in AUD for the year 2020. For the two subcategories, this results in values for 2024 of AUD 6.28 billion direct costs (44.44% of the costs) and AUD 7.85 billion indirect costs (55.56% of the costs) [66] (pp. 1212-1219).

As these values are calculated exclusively for myocardial infarction and stroke, a prevalence-based adjustment is required in accordance with the methodology of

possibility one for case one, as outlined in the theoretical methods chapter. The aggregated share of prevalence of both subcategories in 2021 corresponds to 41.51% [23]. Following the adjustment of the aforementioned values with the specified ratio, the 2020 USD-AUD exchange rate is utilised, which is equivalent to USD 0.69 per AUD [36], to ascertain the appropriate currency for our study. As the values are already based on the year 2024, but are given in AUD for 2020, after conversion to USD for 2020, only the inflationary adjustment using the consumer price index ratio of both years is then required. Overall, this results in a CVD COI of USD 28.49 billion for Australia in 2024, with direct costs amounting to USD 12.66 billion (44.45% of the costs) and indirect costs totalling USD 15.83 billion (55.55% of the costs).

In order to ascertain the health costs of CVD in Iran, two studies are required, the combination of which corresponds to the second possibility of the first case. The first of these determines the COI of people over 60 years of age by applying a prevalence-based approach from a societal perspective. The direct share is calculated using a bottom-up micro-costing method, which determines the medical and non-medical costs. The financial burden of medical care encompasses a wide range of expenses, including hospitalization, rehabilitation, outpatient visits, medical and imaging tests, diagnosis and medications. Non-medical costs encompass transport, food, accommodation and other expenses for patients and carers. The calculation of indirect costs is performed through the utilisation of the human capital approach. These include productivity losses due to the inability to work and caregiving responsibilities. According to this study, a total of USD 1.89 billion in health costs were incurred by individuals over the age of 60 due to CVD in 2021. The majority of these costs were the result of direct expenses. The equivalent figure is USD 1.75 billion (92.90% of the costs). As indirect costs are primarily made up of productivity losses, which in turn are largely made up of the costs of incapacity to work, the proportion is correspondingly low for individuals over the age of 60. In this study, the share totals USD 0.13 billion (7.10% of the costs) [67] (p. 1355). In the theoretical section of the methodology, we have already worked out that both COI shares shift depending on age, which requires an adjustment and different extrapolations to the total population. Consequently, a further study is needed to determine the general relations between the two types of costs. A study is used that ascertains the COI in south-west Iran in 2016. The underlying method is a combination of a top-down prevalence-based approach to calculate the direct costs and a human capital approach, which is supplemented by a friction cost method to determine the productivity losses. The first COI component comprises medical costs, which are further categorised into inpatient and outpatient care, home care, rehabilitation, consultations with specialists and other care services, diagnoses, and medications. Conversely, non-medical costs encompass expenses related to transport, relocation, therapies, domestic help, dietary adjustments, house, car, special equipment, travel and accommodation, communication, housekeeping, childcare and related items. Indirect costs are attributed to the loss of resources due to morbidity and mortality [68]. This study focuses on the economic burden for a specific region of Iran, whereas the earlier study examined the COI for the entire population of individuals over 60 years of age in Iran. In our study, the percentage shares of direct and indirect costs in total health costs are utilised. As asserted by Emamgholipour et al. (2018), the direct costs are responsible for 60.00% of the total COI, while the indirect costs account for 40.00% [68]. It is important to note that this study does not look at a specific proportion of the population, but rather, it encompasses the entire patient cohort. To obtain representative values for Iran, it is necessary to consider the prevalence of individuals over the age of 60 in relation to the prevalence of CVD in the overall population. The proportion in question is 39.85% [23], which can be used to estimate the total direct costs in Iran using the resulting quotient. These amounted to USD 4.39 billion in 2021. The simplifying assumption is that direct medical

and non-medical costs are incurred in equal amounts for the total population. In order to correctly reflect the determined ratios of direct and indirect COI, the latter are to be calculated using both shares, which must correspond to 40.00% of the total costs. These amounts are equivalent to USD 2.93 billion, resulting in a total COI of USD 7.32 billion for Iran in 2021. This value is then adjusted for inflation and demographics for the year 2024, emerging in a total value of USD 8.78 billion, direct costs of USD 5.27 billion and indirect costs of USD 3.51 billion.

In order to determine the health costs from the studies for Nigeria, Cameroon and India, it is necessary to calculate the hospitalization rate. This indicates the number of individuals diagnosed with CVD who subsequently present themselves at healthcare institutions for treatment. Given the absence of specific data for African countries, it is necessary to employ an international estimate of the rate. A study by the Australian Heart Foundation assesses the hospitalization rate for CVD at one person per minute [69]. Extrapolating this value to one year and dividing it by the prevalence of CVD in Australia [23] results in an annual hospitalization rate of 18.79%. For the purpose of this study, the values for individual territorial areas per 100,000 inhabitants are given for Canada [70] (pp. 1081-1090). When these percentages are applied to the context of CVD, the hospitalization rate is 8.64%. A study conducted by the German Society for Thoracic, Cardiovascular and Vascular Surgery reports that 1,560,441 patients with heart disease were treated as inpatients [71]. Standardised with the prevalence of CVD in Germany in 2021 [23], this results in a hospitalization rate of 12.31%. A study is available for India that estimates the hospitalization rate of CVD sufferers there at 10.90 million for 2021 [72] (pp. 671-677). When this figure is compared with the corresponding prevalence, the hospitalization rate is 12.23%.

In the course of our study, we simplistically assume a hospitalization rate of ten CVD sufferers per 100 affected persons for Nigeria and Cameroon. It is acknowledged that the estimate for Canada does not include Quebec, which consequently results in a distorted estimation of the hospitalization rate. In Germany, heart disease hospitalization data are collated, yet this information corresponds to a single category. It is anticipated that the Australian and Indian values will provide the most accurate estimations of the actual hospitalization rate. In light of the discourse surrounding the underfunding and substandard management of healthcare systems in African countries [73] (pp. 395-403), it is hypothesised that access to healthcare facilities in these nations is more constrained than in Australia, which is ranked second in the global Overall Health Index. This assertion is substantiated by the observation that these African countries have been ranked 86th (Nigeria) and 149th (Cameroon) in the Overall Health Index [74]. Consequently, it is anticipated that the hospitalization rate in these countries is comparatively lower. As India is ranked 66th in this ranking with a hospitalization rate of 12.23%, which is significantly higher than the African countries, but closer to the African countries compared to Australia, we assume in our study that a hospitalization rate of 10.00% is appropriate for these countries.

In order to determine the COI of CVD in Nigeria, a combination of several studies is required. The systematic approach is analogous to the third possibility of the first case for calculating the COI for partially existing costs. Ipinnimo et al. (2023) assess the direct and indirect costs of non-communicable diseases (NCDs) in private and public healthcare facilities for each patient. An investigation is conducted in Ado-Ekiti, a Nigerian city, in which several hospitals, health centres and other health buildings are examined. The focus is exclusively on private or public patients, excluding those who meet both criteria. The direct costs incurred in this context encompass those for consultation, registration, examinations, accommodation, transport, catering, medication, consumables and other direct payments. The indirect costs result from the human capital approach, which includes loss of income and wages during illness as well as healthcare. The mean monthly direct costs

for patients in private healthcare facilities amount to USD 39.41 per person, while those for patients in public facilities are USD 26.51 per individual. The associated indirect costs are calculated to be USD 4.34 per capita in private facilities and USD 10.39 per patient in public facilities [75] (p. 6). As Ipinnimo et al. (2023) define DM and hypertension, which is a subcategory of CVD, as components of the NCD, a conversion to CVD is required [75] (p. 6). For this purpose, the relevant current prevalences are determined from the GBD Compare tool of the University of Washington. Firstly, the prevalent cases of hypertension are set in relation to the total cases of DM and this disease and then extrapolated for CVD by including the corresponding prevalence data in relative terms. This study provides a comprehensive analysis of the financial burden of CVD for patients seeking treatment in both public and private healthcare facilities across Nigeria. It is now imperative to project the patient costs incurred in healthcare facilities to the total number of CVD patients. Firstly, it is necessary to estimate the total proportion of patients in private and public healthcare facilities. As there is an absence of data for CVD in the context of Nigeria, a further study is utilised to ascertain the proportions for acute coronary events. As these correspond to a subarea of CVD and the study records values across the whole of Nigeria with 21 hospitals, it is assumed that the values determined are representative of our work. As Isezuo et al. (2022) find, between 2013 and 2018, 61.70% of patients were treated in public hospitals, while 38.30% of patients were taken care of in private hospitals [76]. In light of the direct per-patient costs and the prevalence of CVD from 2019, the distribution of patients by healthcare institution and the hospitalization rate of 10.00%, the direct COI for 2019 in Nigeria can now be determined. This equates to USD 639.94 million, with the calculation based on the current distribution of disease burden. To calculate the indirect COI, it is necessary to consider the indirect per-patient costs and the other factors that have been enumerated. In addition, the prevalence of non-retirees as a proportion of the total prevalence of CVD is required. In order to reflect the most current values possible in this ratio, those from 2021 are again used. In Nigeria, the retirement age has been set to be 50 since 2019 [77], which is why only people who are younger are taken into consideration when determining the indirect costs. The corresponding share is 49.19%. As a result of the aforementioned factors, the total indirect costs incurred in Nigeria in 2019 amount to 808.02 million USD due to CVD. The total COI is therefore USD 1.45 billion, of which 44.20% are direct and 55.80% indirect costs. When adjusted for inflation and demographics for the year 2024, the total CVD COI is USD 2.22 billion, of which USD 0.98 billion are attributable to direct costs and 1.24 billion USD to indirect costs.

Subsequent to the extraction of data from studies for which information on both direct and indirect costs is available, studies from which only the direct COI part can be calculated follow, corresponding to the second case of the method for calculating the COI for partial costs.

The first such study provides an analysis of the financial implications of cardiac and cerebrovascular diseases in Argentina, Brazil, El Salvador and Trinidad and Tobago for 2020. This study, which focuses primarily on the costs of sugar-sweetened beverages, quantifies the direct medical costs for the countries mentioned. As there is a study for Brazil that determines the overall COI, the corresponding values from the aforementioned study are omitted. The study utilised for the remaining countries defines the direct medical costs as the sum of expenses for diagnosis, treatment and subsequent actions. Consequently, it can be regarded as a reliable estimate of the direct COI in these countries. The total direct costs amount to USD 3.44 billion, of which USD 2.85 billion (82.91% of the costs) are ascribable to Argentina. El Salvador is the second-highest contributor with USD 0.39 billion (11.37% of the costs), followed by Trinidad and Tobago with USD 0.20 billion (5.72% of the costs) [78]. As the just mentioned costs of CVD in the respective countries are attributable to a combination of cardiac and cerebrovascular diseases, a relative

prevalence adjustment is imperative to ascertain the true burden of CVD. The procedure, therefore, corresponds to the first possibility of the second case of the method for calculating the COI for partially existing costs. These can be found in the GBD Compare tool from the University of Washington, which provides detailed data for this purpose. In this instance, 55.37% of the CVD prevalence in Argentina originates from the two aforementioned subcategories, while the proportions in El Salvador (69.73%) and Trinidad and Tobago (85.11%) are significantly higher [23]. After the extrapolation of values for all cardiovascular diseases, they are adjusted for inflation and demographics for 2024. A prior conversion into USD is not necessary, as the study has already converted the local currencies. This procedure leads to an estimated 7.30 billion USD in direct health expenditures for the three countries in 2024, with Argentina accounting for the largest share of USD 6.32 billion (86.60% of the costs), followed by El Salvador with USD 0.69 billion (9.48% of the costs) and Trinidad and Tobago with USD 0.29 billion (3.91% of the costs).

In order to ascertain the COI of CVD in Cameroon, a study is employed that estimates it using a prevalence-based, bottom-up, micro-costing method. The direct per-patient costs are determined from a healthcare system perspective, while any indirect costs were not considered. The system of the third possibility of the second case is utilised for the calculation of the COI. In this study, the health costs for IHD, ischaemic stroke, haemorrhagic stroke and hypertensive heart disease (HHD) are ascertained through the analysis of data from 850 patients across two of the largest hospitals from 2013 to 2017. This results in annual costs of USD 1.22 million for all 850 patients, corresponding to a direct COI of USD 1441.08 per patient for the four subcategories [79] (p. 140). To extrapolate this value to CVD as a whole, current prevalence figures for the individual subgroups are required. The prevalence of IHD, HHD, ischaemic and haemorrhagic stroke as a proportion of CVD is therefore 62.66% [23]. This percentage is utilised to estimate the per-patient costs of CVD under current disease burden ratios. By multiplying the value by the prevalence of CVD from 2017, the hospitalization rate, the inflation rate and the corresponding population adjustments, the representative value for 2024 is obtained. The direct COI of CVD for 2024 in Cameroon is thus determined to be USD 610.34 million.

The penultimate country for which primary data are available is India. Here, a study is conducted with the objective of ascertaining the direct costs incurred by patients treated in a super-speciality hospital between April 2017 and June 2018. A total of 100 medical reports are included in the study, and the number of a specific subcategory is determined using a prevalence-based approach. As illustrated in Table S1, a substantial number of cost factors are taken into account. These include the cost of the equipment, the cost of the building and the overhead expenses. Salary, hospital supplies and investigation are also considered [80].

Table S1. Direct cost components of the cost-of-illness of cardiovascular diseases in India

Source: Kumar et al. (2022) [80]

CAPITAL COSTS	OPERATIONAL COSTS
**Cost of Equipment**	**Salary**
**Cost of Building**	Faculty
**Overhead Cost**	Residents
Electricity	Nursing Staff
Laundry services	Technical staff
Air-conditioning	Hospital Attendant
Central Sterile Supply Department (CSSD)	Sanitary Attendant
Manifold services	Security Guard
Bio-Medical waste management	**Overhead Costs**
Dietary services	Electricity
Manifold services	Laundry services
Bio-Medical waste management	Air-conditioning
Dietary services	CSSD
Manifold services	Manifold services
	Bio-Medical waste management
	Dietary services
	Maintenance cost
	**Hospital supplies**
	Surgical store
	Medical store
	General store
	Linen store
	Stationary store
	**Investigation**
	Laboratory Investigation
	Radiological Investigation

When all the relevant factors are taken into account, the per-patient cost of CVD in India is USD 3,842 [80]. To calculate the total direct COI for 2018 and for 2024, the prevalence of CVD from the first year [23] and a hospitalization rate that is as up to date as possible are required. The study mentioned above is utilised for this purpose, as it estimates the number of hospitalizations for 2021 at 10.90 million [72], from which the corresponding hospitalization rate can be calculated. While a globally standardised rate of 10.00% was previously assumed for Nigeria and Cameroon, it is 12.23% in India. Utilising the aforementioned data in conjunction with the 2018 prevalence figures, the direct COI of CVD for 2018 can be calculated employing current disease burden ratios. This value must then be adjusted for inflation and demographics for 2024. Following the application of all relevant modifications, the current direct COI for India is USD 50.72 billion.

The first and only possibility of the third case considered here occurs in a study on the economic burden of heart disease for Mexico. Stevens et al. (2016) calculate the total COI for this subgroup of CVD for the year 2015 in Mexican pesos. The direct component corresponds to the financial implications of the impact of heart disease on the healthcare system, whereas the indirect part is associated with factors such as morbidity, premature death, caregiver costs and diminished productivity. Heart disease, as also previously outlined, is a term used to describe a range of medical conditions, including heart failure, myocardial infarction, atrial fibrillation and hypertension. In 2015, the total health costs amounted to 303 billion pesos [81]. University of Washington's GBD Compare tool asserts that the contemporary prevalence of these four subcategories corresponds to 54.84% of those attributable to CVD [23]. When calculating the total health costs of Mexico for the year 2024, it is necessary to include this ratio in the calculation, to convert the currency according to the 2015 MXN-USD exchange rate of MXN 15.87 per USD [36], and then to adjust this value for inflation and demographics. The result of this calculation are total health costs of USD 46.11 billion in 2024 for Mexico.

Prior to the discussion of the data utilised in our study to ascertain the global COI of DM, it is imperative to elucidate the term. DM consists of type 1 diabetes mellitus (T1DM) and T2DM. However, only the latter disease is caused by dietary risk factors.

Nevertheless, given that the DALY rates presented in the subsequent subchapter relate the burden of disease of the individual diseases caused by nutritional risks, which as mentioned only occur in the form of T2DM, to the total burden of disease, which can be extended to DM, it is logical and pragmatic to consider the aggregate costs of DM.

A study published in the American Diabetes Association is utilised in order to ascertain the global economic burden of DM [82] (pp. 963-970). This study determines the costs of DM in 179 countries and territories for 2015 and predicts those for 2030 for different scenarios. The countries and territories employed in this study are consistent with those considered in our analysis. A multi-step procedure is utilised to calculate direct costs. Firstly, it is hypothesised that healthcare expenditure and mortality rates are consistent across age groups and distributions. Based on this, the percentage expenditure of people affected by DM in relation to those not affected by DM is determined by comparing the most extensive possible healthcare expenditure, which includes at least inpatient and outpatient care and the cost of medicines. Finally, the additional costs due to DM are calculated using a cost ratio and the previously examined data. In this study, indirect costs are equivalent to the production losses to the working population and consist of retirement, absenteeism, presenteeism, i.e. reduced productivity while working, and premature death before retirement. The latter is standardised to 65 years. These factors are monetised via average daily or annual wages [82] (pp. 963-970). The procedure and the data collected to determine the economic burden of the individual countries and territories correspond to the methodology of the COI approach. In this instance, all available costs are collated with the objective of obtaining the most accurate estimate of the global monetary burden of the disease. In this study, as in our work, USD is used as the currency, thereby facilitating the direct transfer of the data. The economic burden of DM in countries and territories not included in this study is determined using the score-based comparison described previously. A detailed list of the total costs of DM, the proportion of costs in relation to GDP and the share of indirect costs per country or territory in 2015, as well as in three scenarios in 2030, can be found in the 'DM COI' table in Supplementary Materials File S2. Following the aggregation of the individual values, the total costs of DM of the 179 instances amount to USD 1,317.75 billion. Of this, 856.93 billion USD (65.03%) are direct costs and USD 460.82 billion (34.97%) are indirect costs. To adjust these values to the year 2024, it is necessary to obtain data on the inflation of the USD from 2015 to 2024 and on the population figures for the individual countries and territories for both years. These findings are drawn from the studies presented in the first subchapter.

As Bommer et al. (2018) round the costs to two decimal places [82] (pp. 963-970), no more precise statements are available in this regard. Since these are determined in the format USD x.xx billion, a value of 0.00 may result for small countries and territories. However, given that the share of indirect costs and the ratio to the respective GDP are also specified in addition to the total costs of DM, the formula introduced in the chapter on standardization for offsetting GDP with the share of healthcare costs is used in this case. This procedure is required for the countries Gambia, Guinea-Bissau and Sao Tome and Principe. The current GDP value is utilised, as this allows the costs to be estimated more realistically. The most recent data are available for the year 2023 and is published by the World Bank [46]. The GDP of Gambia is priced at USD 2.40 billion, that of Guinea-Bissau at USD 2.05 billion, and that of Sao Tome and Principe at USD 0.68 billion. These values are now multiplied by the shares of health costs by DM. Thereafter, they are subject to adjustment from 2023 to 2024, a process which is undertaken using inflation data and population statistics.

The following section will now examine in detail the study by Chen et al. (2023), which provides the data basis for the health costs of neoplasms, and which quantifies the total costs of 29 types of cancer from 2020 to 2050 in 204 countries and territories are

utilised [83] (pp. 465-472). The costs of the individual types of cancer are made up of the direct share, which encompasses out-of-pocket payments for treatment and increased private health insurance premiums or public health insurance taxes, and the indirect share. The latter results from reduced labour supply due to mortality and morbidity, which is reflected in falling numbers of people in employment, productivity losses and absenteeism. This finding aligns with the COI approach. In order to establish a valid calculation basis for the subsequent calculation of the health costs of nutrition, a comparison must first be made between the types of neoplasms on which this study is based and those induced by malnutrition. It is important to note that, in total, these neoplasm subcategories do not include six that are contained in the GBD Compare tool. However, as these are not caused by malnutrition, this source is suitable for determining the health costs of nutrition. Moreover, the 204 countries and territories correspond precisely to those considered in our study. The financial outlay is expressed in 2017 INT$ or as a proportion of GDP and aggregated over the 31 years from 2020 to 2050 [83] (pp. 465-472). Furthermore, a detailed list with the total costs of neoplasms in 2017, lower as well as upper bounds and the shares of GDP per country and territory can be found in Supplementary Materials File S2 in the 'Neoplasms COI' table.

As we look at real prices in USD in our study, we use GDP ratios. The procedure corresponds to the methodology already presented in the chapter on standardization for aggregated values over a specific period. When calculating the growth factor, we use information on the total DALYs from 2020 and 2050, as we assume that the economic burden of disease increases as the burden of disease increases. Since we also assume exponential growth, the annual GDP shares can be determined by proceeding with a geometric series. The growth rate is therefore approximately 1.19%. The COI of the 204 countries and territories for the 29 types of cancer for the year 2024, not differentiated according to direct and indirect COI, is calculated by multiplying the share of healthcare costs in the GDP of the respective country or territory by the corresponding GDP per capita [84] and the population in 2024.

S3.3 Input data to determine the burden of disease

As with the system employed to validate the diseases considered in our study, the DALY measure is used to determine the respective burden of disease due to external effects of diet. Given that this is derived from the sum of the YLD and the YLL, it is logical to offset it against COI data. Both measures comprise a direct and an indirect component. In our study, all dietary risk factors from the GBD Compare tool of the University of Washington are taken into account. Of the total of 15 items, five relate to overconsumed dietary components, while ten include underconsumed foods [23]. For each of the 204 countries and territories, the DALY number of the 15 risk factors and the total DALY number of the respective disease are determined and set in relation to CVD, DM and neoplasms. The resultant matrix, which is 612 by 15, contains DALY rates that can be utilised to calculate the COI of the diseases down to the proportional health costs of nutrition. Details can be found in Supplementary Materials File S2 in the table 'DALY rates'.

S4. Dealing with uncertainties and limitations

The following section examines in detail uncertainties and limitations arising from the developed methodology and input data. A detailed explanation is provided on how these are handled in our study.

Firstly, there is some uncertainty regarding the restriction to the three diseases CVD, DM and neoplasms. As mentioned in chapter S3.2, although there are other diet-induced diseases, these account for less than 5% of the total DALYs of dietary risk factors, which is why they are excluded from our study. Whilst the health costs of diet may be underestimated in our study, the inclusion of all diseases is not recommended, as already described in the introduction, due to the difficulty of determining the respective COI and the additional resulting uncertainty when estimating the missing values. Similarly, other nutrition-related diseases such as micronutrient deficiencies are not fully covered in our study. These include, for example, vitamin A, iron and iodine. They can affect children's growth, cognitive abilities, as well as work and academic skills. Additionally, they may result in infections and deaths (85). While the former effects and further psychosocial impacts are difficult to measure, the latter are included in the risk factors in our study. For example, vitamin A is found in fish, fruit and vegetables (86), the over- and underconsumption of which is examined in our study.

Furthermore, it should be noted that both the inflationary and demographic adjustments estimate data from a certain year onwards. While this is done from 2023 inclusive for the former, it is done from 2024 for the latter. It is important to mention that, by their very nature, forecasts are subject to a certain degree of uncertainty with regard to the actual values that will occur.

The primary uncertainty associated with our study pertains to the utilization of disparate sources in the calculation of the COI of CVD for individual countries and territories. As elucidated in the preceding chapter, different studies include different types of costs and differentiate between them to varying extents. For instance, the indirect costs of the 27 EU member states encompass not only productivity losses but also informal care [48] (pp. 4752-4767), while the study on the COI of the six Gulf states exclusively includes the former [50]. In our work, it is hypothesized that this uncertainty surrounding the data used is acceptable. The rationale behind this is attributable to the reasonable usage of disparate methodologies and cost breakdowns across various studies. For example, it is not possible to determine more precisely whether absenteeism in different studies merely refers to the absence of the sick person or also includes, for example, the costs of family members caring for the ill person. Consequently, we assume in our study that the COI of

individual countries and territories is optimally evaluated through the utilization of local data surveys, as these data sources possess the capacity to incorporate specific regional characteristics into the calculation.

The presence of further heterogeneity is attributable to the employment of differing approaches to determining the respective COI. With regard to direct costs, a prevalence-based approach is generally used, but there are also alternative methods of collecting data, such as an incidence-based approach or the use of surveys. Furthermore, the methodologies employed can be classified as top-down or bottom-up. The human-capital approach is frequently applied in the context of indirect costs. However, multiple methodologies for the collection of data exist in this area. In our study, it is again assumed that local studies take into account the peculiarities and characteristics of the countries and territories under consideration when selecting the methodology utilized to calculate COI. It is therefore concluded that differences in data collection in this regard are acceptable.

The heterogeneity of the COI data for the three diseases is counteracted in our study by the methods developed for harmonizing and standardizing the data. Inflation and population data are used as a basis for this. For future research in this area, it is advisable to critically examine whether it makes sense to include additional descriptive factors such as GDP. Furthermore, for a more precise and uniform quantification of disease costs for the countries and territories under consideration, especially for CVD, the same COI approaches and the same types of costs examined are required. For DM and neoplasms, there are studies that determine the costs consistently for a wide range of countries and territories. Due to the importance and influence of CVD, the possibility or necessity of carrying out a correspondingly standardized data collection also arises.

Furthermore, we estimate partial cost data using prevalences. These, in addition to DALYs, deaths and incidences, are determined from the GBD Compare tool mentioned above, which provides corresponding numbers for the year 2021 [23]. The values stated in this study are the mean of a range, which can vary in terms of scope depending on the country and territory in question. This finding suggests a certain degree of flexibility in the data. We address this issue in our study by conducting a scenario analysis of the resulting values, taking this range into account. In addition, the assumption of the direct scalability of costs through prevalence is subject to uncertainties. In our study, the values for the year 2024 are calculated, whereas the prevalence data currently available originates from the year 2021. Consequently, the projections do not reflect changes that may have occurred between the two years. As no more recent data are accessible, it can be assumed that it reflects the current situation. Therefore, the prevalences from 2021 are utilized.

Determining the COI of DM in 179 countries and territories is also subject to dubiety. One of the main uncertainties in this regard has previously been referenced in the explanation of the scaling of the indirect costs of Nigeria. As Bommer et al. (2018) determine the costs for individuals aged between 20 and 79 years [82] (pp. 963-970), it is necessary to extrapolate these figures to the total population. This extrapolation to all age groups is only conducted for the direct costs, but not for the indirect costs. The reason for this is the presumption that direct costs are incurred by everyone who falls ill, while indirect costs are largely borne by those in employment. It is therefore assumed that indirect costs only begin at the age of twenty and continue until the age of seventy-nine.

Furthermore, the financial burden of the countries for which the rounding of the study results in costs of USD0.00 billion is determined using the ratio of health costs to the respective GDP from 2015. The reason for this is that although the costs amount to zero due to rounded figures, they do exist [82] (pp. 963-970) and are therefore calculated using the specified percentage so that they are not lost. This is afterwards multiplied by the most recent GDP data from 2023. In this instance, the calculation is also based on the

premise that utilizing the most recent values is the most effective method of accounting for current conditions.

In determining the COI of neoplasms, the main uncertainty pertains to the assumption of exponential growth and the calculation of the growth factor. The former is logical when considering that the growth of certain factors is estimated by Chen et al. (2023) on the basis of rates that do not suggest a linear increase [83] (pp. 465-472). The sole value employed in this study, which is available separately for both the start year 2020 and the end year 2050, is utilized for the purpose of determination. This is consistent with the DALYs for both years, which are used to calculate the growth factor.

In the interest of enhancing the clarity and coherence of the argument of utilizing latest prevalence data, it would be more intuitive to posit that the costs were incurred in a previous year and, as a result, should be standardized with the proportions of the subcategories at that time. However, this would mean that although the data would be better estimated for past disease burdens, current developments and shifts would not be taken into account. Our approach of offsetting against current ratios is therefore legitimate in the sense that it can be assumed that the costs of diseases do not change depending on their incidence, but rather due to inflation and technology, whereby the latter is challenging to ascertain and thus excluded from the scope of this study. The consequence of this approach is that the calculated values for the base year are not based on the prevalence data at that time and are therefore less representative of the past. However, it also has the consequence that the results reflect contemporary conditions and therefore represent a meaningful scaling for current calculations. It is imperative that the values relate to ongoing developments and circumstances, thereby providing a more accurate estimation of the present situation.

The calculation of the total health costs for per-patient data from a previous year is contingent upon the consideration of absolute prevalence data from that same year. This is predicated on the fact that, after the corresponding calculation, this value is adapted for inflation and demographics to the year 2024. The latter adjustment is carried out using current population data. It can be assumed that as the population increases, the prevalence figures for the respective disease are also likely to rise, without taking technological or medical advances into account. If the present values were used instead of the past prevalence data to convert the per-patient costs, the demographic adjustment would be carried out twice by assumption.

In the event of the calculation being conducted in accordance with this scheme, the COI for the previous year is obtained with contemporary sickness load ratios. However, an inflationary and demographic adjustment is required to recalibrate this value for the year 2024.

Since the study to estimate the COI in Iran calculates it for people over the age of 60, a group which, by definition, has lower indirect costs than younger generations, it is necessary to use general ratios to scale these values. The determination of these metrics is conducted by a comprehensive survey of the health cost distribution of coronary heart diseases, a subcategory of CVD, in south-west Iran. Given that the underlying disease does not correspond to the entirety of CVD and a part of Iran is considered, there is a certain uncertainty regarding the representativeness of the values. Nevertheless, it can be assumed that the distribution of the costs of this subcategory, which accounted for over half (57.64%) of the prevalence of CVD in Iran in 2021 [23], is representative of CVD. In addition, different age groups are considered in this study, which means that general trends can be identified, although only a subregion of the country is analyzed.

When calculating the direct health costs of Nigeria, Cameroon and India, a hospitalization rate is utilized in each instance to estimate the proportion of sick people who visit healthcare facilities. Although relevant data are available for India, an estimate of the

hospitalization rate is required for the two African countries in question. Various comparative values were used to determine this. Based on the Overall Health Index and the fact that the healthcare systems in Africa are relatively poorly funded, it was assumed that there is a hospitalization rate of 10.00% for CVD in Nigeria and Cameroon. We include the ranking based on the aforementioned index and presume that hospitalization is lower in countries with poorer healthcare systems. The value of 10.00% is therefore subject to uncertainties regarding the actual, country-specific level of hospitalization.

In the calculation of the indirect COI for Nigeria, the total number of individuals below retirement age who are afflicted with illness is utilized. This implicitly assumes that children and young people can also suffer productivity losses, which can only be incurred through engagement in paid employment. Figures from the International Labour Organization (ILO) in collaboration with the National Bureau of Statistics of Nigeria prove that this theory is indeed valid in this particular instance. The data show that over half of the children in Nigeria are involved in economic activities, with 39.2% of children participating in child labor [87]. In contrast to the prevalence-based adjustment of the partial study to determine the COI of DM, it is therefore assumed that the use of the prevalence of all age groups below retirement is appropriate. Conversely, in the case of DM, only the direct costs are adjusted by prevalence for the total population, but not the indirect costs. The underlying rationale for these disparate approaches is predicated on the scope of the respective studies. While the Nigerian COI covers one country where child labor is pervasive, the study by Bommer et al. (2018) encompasses 179 countries and territories, incorporating data from individuals aged 20 to 79 [82] (pp. 963-970). In consideration of the global context, where the prevalence of child labor is considerably lower than in Nigeria [87,88], it is not feasible to presume its uniform presence and relative intensity across all regions. Consequently, the assumption of indirect costs below 20 years is not made when transferring the study on the costs of DM.

Furthermore, the calculation of the CVD COI in Nigeria exclusively includes individuals below the retirement age of 50. To comprehend the phenomenon of productivity losses, it is imperative to examine the definition of such losses, which says that they correspond to the market value of the individual's future contribution to production if they continue to work in full health [18] (pp. 327-337). When this is taken into consideration, in conjunction with the average life expectancy in Nigeria of 63.4 years [89], the assumption made regarding the actual indirect costs appears to be substantiated.

In order to calculate the financial burden of CVD in India, the per-patient costs of a super-speciality hospital are used. The All India Institute of Medical Sciences in New Delhi is a preeminent healthcare facility in the country, equipped to meet all the requisite criteria for the treatment of CVD [80]. This raises a question of the representativeness of the data, given the assumption that the treatment methods and costs observed at this facility differ from those at other healthcare facilities with less extensive resources, which are not included in the study. This uncertainty is acknowledged, yet it is hypothesized that certain costs are elevated relative to other facilities, potentially attributable to the adoption of contemporary technologies. Conversely, costs emanating from the utilization of these technologies, such as those associated with rationalization, are conjectured to be diminished.

When estimating missing, direct COI values, the PPP-adjusted per-capita health expenditure and per capita GDP of the individual countries and territories from 2021 are used in addition to the burden of disease indicators that have been previously outlined. As with DALYs, deaths, incidence and prevalence, it is assumed that although these current values do not include the developments from 2021 to 2024, they are nevertheless representative for a comparison, as they represent the latest data situation.

The weighting of these factors is also subject to uncertainties when calculating the scores for the country-specific comparison. They are selected in our study because, in combination with the scale factor, they engender minimal deviations during the estimation of available studies' values. It is considered sufficient to form the subsequent distance matrix by rounding the values to one decimal place. This uncertainty also exists in the burden of disease factor for the scale factor. The combination of population, healthcare expenditure and burden of disease also implies a further vagueness. Despite the fact that this factor is continually compared with numerous others in the course of our study and is invariably shown to provide the most accurate estimate for reference data, it is possible that another factor or combination of factors may be found that can produce an estimate that is even more precise.

In calculating the burden of disease, the COI values of CVD, DM and neoplasms are offset against DALY rates, which determine the proportion of DALYs due to a risk factor in the form of a disease in the total DALYs of this disease. The resulting uncertainty is the use of DALYs, whereas previously prevalences were employed in the calculation of the COI. However, this calculation is logical if one takes into consideration the basis upon which it is calculated. The COI is generated both directly and indirectly by those suffering from the disease in question. The total number of cases is directly proportional to the prevalence, and thus the costs of the disease are directly proportional to this number. The utilization of prevalence as a metric for determining the COI is substantiated by the predominance of studies that employ this indicator as the foundation for their calculations [18] (pp. 327-337).

In the subsequent calculation of diet-induced health costs, however, the use of DALYs is more appropriate. The COI is invariably calculated for a period of one year, in our study for the year 2024. According to the established definition, the DALYs represent the years of life spent with an illness and those lost due to premature death. In this instance, too, a direct and indirect proportion of life years can be recognized to a certain extent. The direct and indirect costs of an illness per year are therefore offset against the direct and indirect years of life lost due to the same condition. This illustration elucidates the rationale behind the utilization of DALYs instead of prevalences in the final step of determining nutrition-induced health costs.

Notwithstanding the fact that the minimum of the disease indicators is invariably zero in the GBD Compare tool, a small number of data downloads result in negative values. As negative DALYs would imply an inversion of the actual statement of disease-adjusted or lost life years due to disease and would therefore represent de facto life years gained, the negative values in our study are zeroed out. This phenomenon can be illustrated by the overconsumption of red meat in certain countries and territories. Conversely, negative DALYs and, by extension, negative costs would signify income from malnutrition, a notion that is not only illogical but also contradicts established principles of economic analysis.

S5. Discussion - Additional information

In the following, the dietary health costs of the three diseases CVD, DM and neoplasms determined in our study are evaluated in detail against the background of further studies and their correlation with DALYs and GDP is investigated.

Of the total costs of USD 4,794.33 billion due to CVD, DM and neoplasms in 2024, USD 2,162.79 billion are due to CVD. A comparison of this figure with estimates from the World Heart Federation reveals that the former is higher. While the costs according to this study will amount to USD 1,044.00 billion in 2030, of which 55.00% will be direct and 45.00% indirect costs [90], the economic burden of disease calculated in our study is higher. Conversely, the American Heart Association estimates that the costs in the USA

alone will be equivalent to USD 1,100.00 billion in 2035 [91]. Assuming that the share of CVD costs in the global economic burden remains constant at 35.70%, this results in global health costs totalling USD 3,081.63 billion. Utilising the database on average USD inflation, which estimates consumer price indices until 2029, and presuming that these will grow at the same rate from 2029 to 2035 as from 2028 to 2029, this results in total inflation of 26.72% from 2024 to 2035. Adjusting the projected health costs by this factor results in costs of CVD of USD 2,451.11 billion for 2024. As we have ignored the corresponding demographic changes, we can conclude that the value we have calculated is in a plausible range.

An examination of the global economic burden of DM reveals that, according to the findings of Bommer et al. (2018) and our own estimates for the countries and territories lacking data, the global cost for 2024 amounts to USD 1,982.18 billion [82] (pp. 963-970). Of this, USD 1,337.69 billion is attributable to the direct share. A comparison of the latter figure with the International Diabetes Federation's estimate of USD 1,030.00 billion for 2030 suggests the possibility of overestimation [92]. However, given that the just mentioned study exclusively limits direct costs to healthcare expenditure, while other factors are generally also included in the COI of diseases, it can be assumed that the actual direct COI of DM is higher. It is also noted that the study by Bommer et al. (2017) [93] (pp. 423-430) provides estimates with higher specificity [92]. As the authors of the study of Bommer et al. (2018) utilised in our paper to calculate the economic burden of DM also largely comply with the authors of the 2017 study, and the values employed for the calculations in the 2018 study are also calculated for the first time in the 2017 study, it can be assumed that the determined values for 2024 are plausible with regard to the amount.

Chen et al. (2023) assess the costs of 29 types of cancer for 204 countries and territories [83] (pp. 465-472). It is not necessary to undertake exhaustive validation of this study, as it embodies the prevailing standard to which all reports, studies and journals refer.

The relationship between DALYs as well as GDP and health costs is explained in detail below.

If we look at the correlation between DALYs and the COI for CVD (r = 0.32), DM (r = 0.51) and neoplasms (r = 0.59), which is shown exemplarily for DM inFigure S1, it becomes apparent that an increase in DALYs is generally associated with higher health costs. Corresponding illustrations for the other two diseases can be found in the 'Supplementary Figures' table in Supplementary Materials File S2. It is particularly noteworthy that the USA does not have the highest number of DALYs for all three diseases, but it does have the highest costs. For instance, CVD in the USA is responsible for around 17.76 million DALYs and USD 773.54 billion in health costs, whereas in China, it is 100.21 million DALYs and USD 95.52 billion in health costs. For DM, there are 5.07 million DALYs and USD 721.01 billion in health costs in the USA, while in India, there are USD 32.65 billion in health costs for 13.67 million DALYs. This anomaly has also been observed in cases of neoplasms.

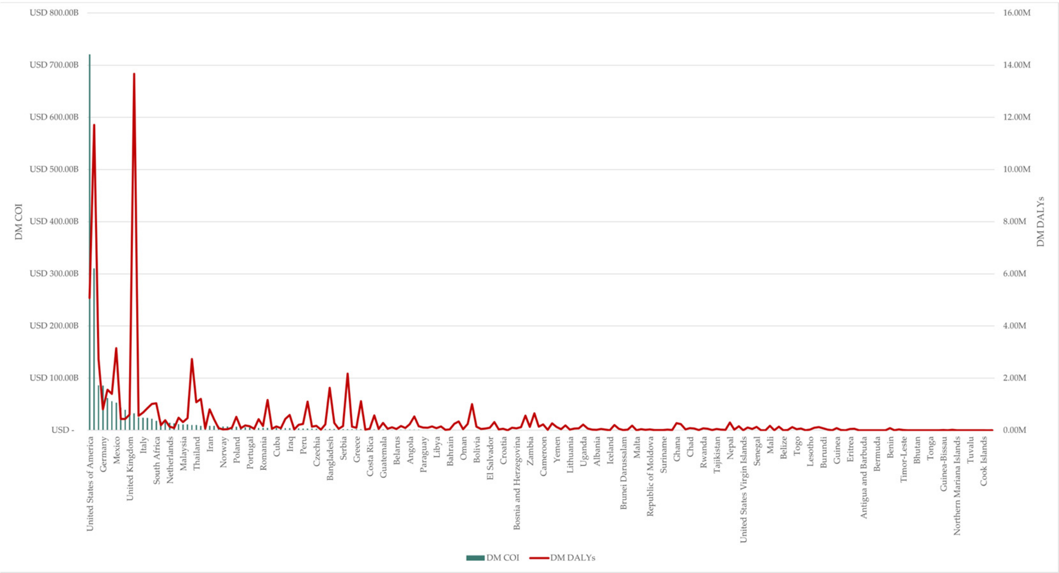

Figure S1. Total health costs and DALYs of diabetes mellitus per country and territory for 2024.

A comparison of the correlation between the health costs of diseases in the countries and territories with GDP reveals a significantly higher correlation than that observed for COI and DALYs, with a concomitant reduction in the number of outliers, as can exemplarily be seen in Figure S2for DM. As before, corresponding illustrations for the other two diseases can be found in Supplementary Materials File S2in the 'Supplementary Figures' table. The correlation coefficient for CVD is 0.89, and 0.97 for DM and neoplasms. This means that it can be assumed that higher prosperity also leads to higher health costs, while higher numbers of DALYs in principle indicate that the costs may also be higher, but this does not necessarily have to be the case.

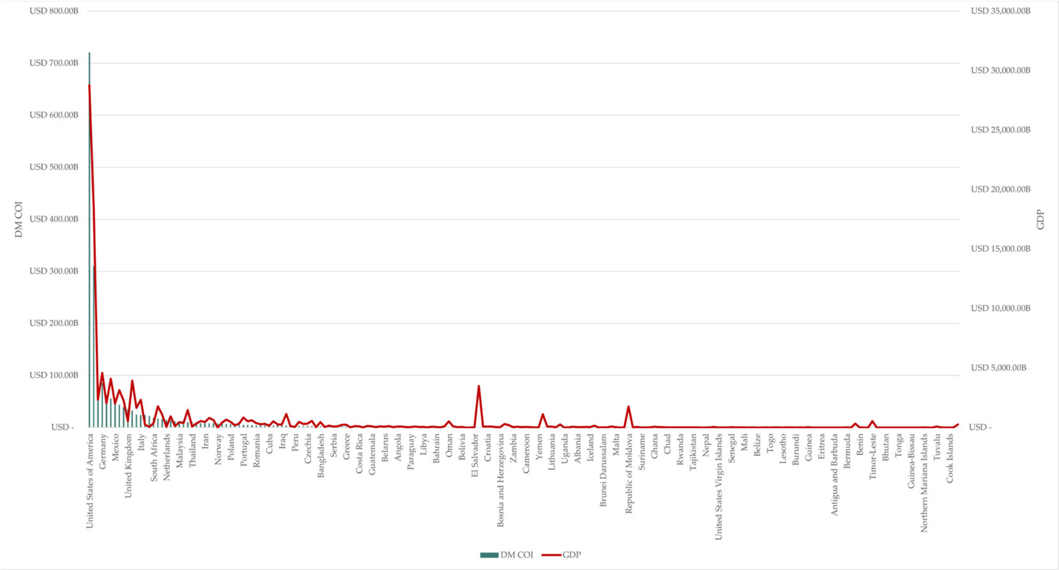

Figure S2. Total health costs and GDP of diabetes mellitus per country and territory for 2024.

References

18. Jo, C. Cost-of-illness studies: Concepts, scopes, and methods. Clin. Mol. Hepatol. 2014, 20, 327–337. https://doi.org/10.3350/cmh.2014.20.4.327.
23. Institute for Health Metrics and Evaluation. *GBD Compare | IHME Viz Hub*; University of Washington: Seattle, WA, USA, 2025. Available online: https://vizhub.healthdata.org/gbd-compare/ (accessed on 18 May 2025).
36. Board of Govenors of the Federal Reserve System. Federal Reserve Board—Foreign Exchange Rates—H.10—February 10, 2025. The Federal Reserve. 2025. Available online: https://www.federalreserve.gov/releases/h10/hist/dat00_al.htm (accessed on 11 May 2025).
37. Antweiler, W. PACIFIC Exchange Rate Service. Foreign Currency Units per 1 Canadian Dollar, 1950–2023. 2024. Available online: https://fx.sauder.ubc.ca/etc/CADpages.pdf (accessed on 25 May 2025).
38. Exchanges Rate UK. Chilean Peso to US Dollar Spot Exchange Rates for 2015. 2025. Available online: https://www.exchangerates.org.uk/CLP-USD-spot-exchange-rates-history-2015.html (accessed on 25 May 2025).
39. Exchanges Rate UK. Colombian Peso to US Dollar Spot Exchange Rates for 2015. 2025. Available online: https://www.exchangerates.org.uk/COP-USD-spot-exchange-rates-history-2015.html (accessed on 25 May 2025).
40. Macrotrends LLC. Euro Dollar Exchange Rate (EUR USD)—Historical Chart. 2025. Available online: https://www.macrotrends.net/2548/euro-dollar-exchange-rate-historical-chart (accessed on 25 May 2025).
41. International Monetary Fund. World Economic Outlook Database, October 2024. 2024. Available online: https://www.imf.org/en/Publications/WEO/weo-database/2024/October (accessed on 11 May 2025).
42. World Bank Group. Population, Total. All Countries and Economies. 2025. Available online: https://data.worldbank.org/indicator/SP.POP.TOTL?end=2021&start=1960 (accessed on 11 May 2025).
43. Worldometer. Population by Country (2025)—Worldometer. 2025. Available online: https://www.worldometers.info/world-population/population-by-country/ (accessed on 25 May 2025).
44. Department of Economic and Social Affairs. World Population Prospects 2024. United Nations. 2024. Available online: https://population.un.org/wpp/ (accessed on 11 May 2025).
45. World Bank Group. Current Health Expenditure Per Capita (Current US$). 2024. Available online: https://data.worldbank.org/indicator/SH.XPD.CHEX.PC.CD (accessed on 25 May 2025).
46. World Bank Group. World Development Indicators. 2025. Available online: https://databank.worldbank.org/indicator/NY.GDP.PCAP.CD/1ff4a498/Popular-Indicators (accessed on 25 May 2025).
47. International Labour Organization. Statistics on Labour Productivity. 2024. Available online: https://ilostat.ilo.org/topics/labour-productivity/ (accessed on 25 May 2025).
48. Luengo-Fernandez, R.; Walli-Attaei, M.; Gray, A.; Torbica, A.; Maggioni, A.P.; Huculeci, R.; Bairami, F.; Aboyans, V.; Timmis, A.D.; Vardas, P.; et al. Economic burden of cardiovascular diseases in the European Union: A population-based cost study. *Eur. Heart J.* **2023**, *44*, 4752–4767. https://doi.org/10.1093/eurheartj/ehad583.
49. Wilkins, E.; Wilson, L.; Wickramasinghe, K.; Bhatnagar, P.; Leal, J.; Luengo-Fernandez, R.; Burns, R.; Rayner, M.; Townsend, N. *European Cardiovascular Disease Statistics 2017*; European Heart Network: Brussels, Belgium, 2017.
50. Elmusharaf, K.; Grafton, D.; Jung, J.S.; Roberts, E.; Al-Farsi, Y.; Al Nooh, A.A.; Bin Belaila, B.; ElShamy, A.; Al-Zuabi, H..; Al Mutawa, K.A.; et al. The case for investing in the prevention and control of non-communicable diseases in the six countries of the Gulf Cooperation Council: An economic evaluation. *BMJ Glob. Health* **2022**, *7*, e008670. https://doi.org/10.1136/bmjgh-2022-008670.
51. Rittiphairoj, T.; Reilly, A.; Reddy, C.L.; Barrenho, E.; Colombo, F.; Atun, R. The State of Cardiovascular Disease in G20+ Countries. Health Systems Innovation Lab, Harvard University, May 2022. https://doi.org/10.54111/0001/HSIL/cvdg20.
52. Mendoza-Herrera, K.; Pedroza-Tobías, A.; Hernández-Alcaraz, C.; Ávila-Burgos, L.; Aguilar-Salinas, C.A.; Barquera, S. Attributable Burden and Expenditure of Cardiovascular Diseases and Associated Risk Factors in Mexico and other Selected Mega-Countries. *Int. J. Environ. Res. Public Health* **2019**, *16*, 4041. https://doi.org/10.3390/ijerph16204041.
53. Agency for Healthcare Research and Quality. Medical Expenditure Panel Survey Home. 2025. Available online: https://meps.ahrq.gov/mepsweb/ (accessed on 25 May 2025).
54. Kazi, D.S.; Elkind, M.S.V.; Deutsch, A.; Dowd, W.N.; Heidenreich, P.; Khavjou, O.; Mark, D.; Mussolino, M.E.; Ovbiagele, B.; Patel, S.S.; et al. Forecasting the Economic Burden of Cardiovascular Disease and Stroke in the United States Through 2050: A Presidential Advisory From the American Heart Association. *Circulation* **2024**, *150*, e89–e101. https://doi.org/10.1161/CIR.0000000000001258.

55. Yang, L.; Wu, M.; Cui, B.; Xu, J. Economic burden of cardiovascular diseases in China. *Expert Rev. Pharmacoeconomics Outcomes Res.* **2008**, *8*, 349–356. https://doi.org/10.1586/14737167.8.4.349.
56. Walker, I.F.; Garbe, F.; Wright, J.; Newell, I.; Athiraman, N.; Khan, N.; Elsey, H. The Economic Costs of Cardiovascular Disease, Diabetes Mellitus, and Associated Complications in South Asia: A Systematic Review. *Value Health Reg. Issues* **2018**, *15*, 12–26. https://doi.org/10.1016/j.vhri.2017.05.003.
57. Tarride, J.-E.; Lim, M.; DesMeules, M.; Luo, W.; Burke, N.; O'Reilly, D.; Bowen, J.; Goeree, R. A review of the cost of cardiovascular disease. *Can. J. Cardiol.* **2009**, *25*, e195–202. https://doi.org/10.1016/s0828-282x(09)70098-4.
58. The Public Health Agency of Canada. Economic Burden of Illness in Canada, 2010. 2017. Available online: https://www.canada.ca/en/public-health/services/publications/science-research-data/economic-burden-illness-canada-2010.html (accessed on 25 May 2025).
59. Kontsevaya, A.; Kalinina, A.; Oganov, R. Economic Burden of Cardiovascular Diseases in the Russian Federation. *Value Health Reg. Issues* **2013**, *2*, 199–204. https://doi.org/10.1016/j.vhri.2013.06.010.
60. Lakić, D.; Tasić, L.; Kos, M. Economic burden of cardiovascular diseases in Serbia. *Vojnosanit. Pregl.* **2014**, *71*, 137–143. https://doi.org/10.2298/vsp1402137l.
61. Balbay, Y.; Gagnon-Arpin, I.; Malhan, S.; Öksüz, M.E.; Sutherland, G.; Dobrescu, A.; Villa, G.; Ertuğrul, G.; Habib, M. Modeling the burden of cardiovascular disease in Turkey. *Anatol. J. Cardiol.* **2018**, *20*, 235–240. https://doi.org/10.14744/AnatolJCardiol.2018.89106.
62. Rosemann, T.; Bachofner, A.; Strehle, O. Kardiovaskuläre Erkrankungen in der Schweiz—Prävalenz und Versorgung. *Praxis* **2024**, *113*, 57–66.
63. Jin, E. Cost of Silent Risk Factors for Cardiovascular Disease in Asia. Saw Swee Hock School of Public Health. 2018. Available online: https://sph.nus.edu.sg/blog/2018/12/18/cost-of-silent-risk-factors-for-cardiovascular-disease-in-asia/ (accessed on 25 May 2025).
64. The Economist Intelligence Unit Limited. The Cost of Silence. Cardiovascular Disease in Asia. 2018. Available online: https://impact.economist.com/projects/ageing-shift/wp-content/uploads/2020/07/The_cost_of_silence.pdf (accessed on 11 May 2025).
65. Stevens, B.; Verdian, L.; Tomlinson, J.; Zegenhagen, S.; Pezzullo, L. PM021 The Economic Burden of Heart Diseases in Colombia. *Glob. Heart* **2016**, *11*, e73–e74. https://doi.org/10.1016/j.gheart.2016.03.257.
66. Marquina, C.; Talic, S.; Vargas-Torres, S.; Petrova, M.; Abushanab, D.; Owen, A.; Lybrand, S.; Thomson, D.; Liew, D.; Zomer, E.; et al. Future burden of cardiovascular disease in Australia: Impact on health and economic outcomes between 2020 and 2029. *Eur. J. Prev. Cardiol.* **2022**, *29*, 1212–1219. https://doi.org/10.1093/eurjpc/zwab001.
67. Tasavon Gholamhoseini, M.; Arjomand Kermani, S.; Yazdi-Feyzabadi, V.; Goudarzi, R. Economic burden of cardiovascular diseases among elderly patients in Iran: A case from a developing country. *BMC Health Serv. Res.* **2024**, *24*, 1355. https://doi.org/10.1186/s12913-024-11808-0.
68. Emamgholipour, S.; Baba Akbari, A.; Pakdaman, M.; Geravandi, S. Economic Burden of Cardiovascular Disease in the Southwest of Iran. *Int. Cardiovasc. Res. J.* **2018**, *12*, 1–6.
69. National Heart Foundation of Australia. Key Statistics: Cardiovascular Disease. 2025. Available online: https://www.heartfoundation.org.au/your-heart/evidence-and-statistics/key-stats-cardiovascular-disease (accessed on 25 May 2025).
70. Botly, L.C.P.; Lindsay, M.P.; Mulvagh, S.L.; Hill, M.D.; Goia, C.; Martin-Rhee, M.; Casaubon, L.K.; Yip, C.Y.Y. Recent Trends in Hospitalizations for Cardiovascular Disease, Stroke, and Vascular Cognitive Impairment in Canada. *Can. J. Cardiol.* **2020**, *36*, 1081–1090. https://doi.org/10.1016/j.cjca.2020.03.007.
71. Deutsche Gesellschaft für Thorax-, Herz- und Gefäßchirurgie. Fakten-Highlights zum Deutschen Herzbericht 2022. 2023. Available online: Dossier_Fakten-Highlights_Deutscher-Herzbericht-2022_DHS_DGK_DGTHG_DGPK_DGPR_2023-09-21_Final.pdf (accessed on 25 May 2025).
72. Patel, S.; Ram, F.; Patel, S.K.; Kumar, K. Cardiovascular diseases and health care expenditure (HCE) of inpatient and outpatient: A study from India Human Development Survey. *Clin. Epidemiol. Glob. Health* **2020**, *8*, 671–677. https://doi.org/10.1016/j.cegh.2019.12.024.
73. Oleribe, O.O.; Momoh, J.; Uzochukwu, B.S.; Mbofana, F.; Adebiyi, A.; Barbera, T.; Taylor-Robinson, W.R. Identifying Key Challenges Facing Healthcare Systems In Africa And Potential Solutions. *Int. J. Gen. Med.* **2019**, *12*, 395–403. https://doi.org/10.2147/IJGM.S223882.

74. NTI; Brown University School of Public Health Pandemic Center; Economist Impact. Global Health Security Index. 2023. Available online: Global Health Security Index (accessed on 25 May 2025).
75. Ipinnimo, T.M.; Elegbede, O.E.; Durowade, K.A.; Adewoye, K.R.; Ibirongbe, D.O.; Ajayi, P.O.; Sanni, T.A.; Fatunla, O.A.T.; Ippinnimo, M.T.; Ibikunle, A.I. Cost of illness of non-communicable diseases in private and public health facilities in Nigeria: A qualitative and quantitative approach. *Pan Afr. Med. J.* **2023**, *44*, 6. https://doi.org/10.11604/pamj.2023.44.6.35494.
76. Isezuo, S.; Sani, M.U.; Talle, A.; Johnson, A.; Adeoye, A.-M.; Ulgen, M.S.; Mbakwem, A.; Ogah, O.; Edafe, E.; Kolo, P.; et al. Registry for Acute Coronary Events in Nigeria (RACE-Nigeria): Clinical Characterization, Management, and Outcome. *J. Am. Heart Assoc.* **2022**, *11*, e020244. https://doi.org/10.1161/JAHA.120.020244.
77. International Social Security Association. Pensionable Ages. 2025. Available online: https://www.issa.int/de/databases/country-profiles/pensionable-ages (accessed on 25 May 2025).
78. Alcaraz, A.; Bardach, A.E.; Espinola, N.; Perelli, L.; Rodriguez Cairoli, F.; La Foucade, A.; de Mello Vianna, C.M.; Guevara, G.; Gittens-Baynes, K.-A.; Johns, P.; et al. Health and economic burden of disease of sugar-sweetened beverage consumption in four Latin American and Caribbean countries: A modelling study. *BMJ Open* **2023**, *13*, e062809. https://doi.org/10.1136/bmjopen-2022-062809.
79. Aminde, L.N.; Dzudie, A.; Mapoure, Y.N.; Tantchou, J.C.; Veerman, J.L. Estimation and determinants of direct medical costs of ischaemic heart disease, stroke and hypertensive heart disease: Evidence from two major hospitals in Cameroon. *BMC Health Serv. Res.* **2021**, *21*, 140. https://doi.org/10.1186/s12913-021-06146-4.
80. Kumar, A.; Siddharth, V.; Singh, S.I.; Narang, R. Cost analysis of treating cardiovascular diseases in a super-specialty hospital. *PLoS ONE* **2022**, *17*, e0262190. https://doi.org/10.1371/journal.pone.0262190.
81. Stevens, B.; Pezzullo, L.; Verdian, L.; Tomlinson, J.; Zegenhagen, S. PM019 The Economic Burden of Heart Diseases in Mexico. *Glob. Heart* **2016**, *11*, e72–e73. https://doi.org/10.1016/j.gheart.2016.03.255.
82. Bommer, C.; Sagalova, V.; Heesemann, E.; Manne-Goehler, J.; Atun, R.; Bärnighausen, T.; Davies, J.; Vollmer, S. Global Economic Burden of Diabetes in Adults: Projections From 2015 to 2030. *Diabetes Care* **2018**, *41*, 963–970. https://doi.org/10.2337/dc17-1962.
83. Chen, S.; Cao, Z.; Prettner, K.; Kuhn, M.; Yang, J.; Jiao, L.; Wang, Z.; Li, W.; Geldsetzer, P.; Bärnighausen, T.; et al. Estimates and Projections of the Global Economic Cost of 29 Cancers in 204 Countries and Territories From 2020 to 2050. *JAMA Oncol.* **2023**, *9*, 465–472. https://doi.org/10.1001/jamaoncol.2022.7826.
84. World Population Review. GDP Ranked by Country. 2024. Available online: https://worldpopulationreview.com/countries/by-gdp (accessed on 25 May 2025).
85. Yu, Y.; Li, H.; Hu, N.X.; Wu, X.H.; Huang, X.Y.; Lin, H.T.; Yu, K.L.; Li, J.L. Global burden and health inequality of nutritional deficiencies from 1990 to 2019. *Front. Nutr.* **2024**, *11*, 1470713. https://doi.org/10.3389/fnut.2024.1470713.
86. National Institutes of Health. Vitamin A and Carotenoids. 2025. Available online: https://ods.od.nih.gov/factsheets/VitaminA-Consumer (accessed on 10 January 2026).
87. International Labour Organization; Nigeria Bureau of Statistics. *Nigeria Child Labour Survey 2022*; International Labour Office: Geneva, Switzerland, 2024. Available online: https://labordoc.ilo.org/discovery/delivery/41ILO_INST:41ILO_V2/12129155920002676 (accessed on 23 May 2025).
88. UNICEF. What is Child Labour? 2025. Available online: https://www.unicef.org/protection/child-labour (accessed on 23 May 2025).
89. World Health Organization. The Global Health Observatory. Global Health Estimates: Life Expectancy and Leading Causes of Death and Disability. 2025. Available online: https://www.who.int/data/gho/data/themes/mortality-and-global-health-estimates/ghe-life-expectancy-and-healthy-life-expectancy (accessed on 25 May 2025).
90. World Heart Federation. *Why Circulatory Health Matters*; World Heart Federation: Geneva, Switzerland, 2021
91. American Heart Association. Cardiovascular Disease: A Costly Burden for America. Projections Through 2035. 2017. Available online: Cardiovascular Disease Burden Report | American Heart Association (accessed on 25 May 2025).
92. International Diabetes Federation. *IDF Diabetes Atlas*, 10th Ed.; International Diabetes Federation: Brussels, Belgium, 2021. Available online at https://www.diabetesatlas.org (accessed on 25 May 2025).

Disclaimer/Publisher's Note: The statements, opinions and data contained in all publications are solely those of the individual author(s) and contributor(s) and not of MDPI and/or the editor(s). MDPI and/or the editor(s) disclaim responsibility for any injury to people or property resulting from any ideas, methods, instructions or products referred to in the content.
